# Lon protease reprograms cellular physiology of *Streptomyces coelicolor* resulting in enhance antibiotic production

**DOI:** 10.3389/fmicb.2026.1789434

**Published:** 2026-03-16

**Authors:** Aslı Bayraktar, Sude Kara, Zeynep Demir, Sezer Okay, Sedef Tunca

**Affiliations:** 1 Department of Molecular Biology and Genetics, Faculty of Science, Gebze Technical University, Gebze, Kocaeli, Türkiye; 2 Vaccine Institute, Hacettepe University, Altındaǧ, Ankara, Türkiye

**Keywords:** Lon protease, antibiotic, morphological differentiation, *Streptomyces coelicolor*, transcriptome

## Abstract

The genus *Streptomyces* is widely recognized as a rich source of natural compounds, including antibiotics, immunosuppressants, and herbicides. Synthesis of secondary metabolites is initiated by cellular differentiation and is a complex process regulated by intracellular and extracellular signals, as well as numerous regulatory proteins. ATP-dependent Lon protease plays a key role in cellular proteostasis and stress adaptation. Overexpression of this protease has been shown to increase the production of actinorhodin (ACT) and undecylprodigiosin (RED) in *Streptomyces coelicolor*. However, the systems-level mechanisms underlying this phenotype remain unclear. In this study, we employed a multifaceted approach encompassing whole-genome sequencing (WGS), transcriptomics, and high-resolution imaging to analyze how Lon reprograms the cellular physiology of the hyper-antibiotic-producer recombinant strain, Sco-pRA*lon*. WGS revealed that the pRA*lon* recombinant vector, which has a ΦC31 *int/attP* site, integrates not only at the canonical *attB* site within SCO3798 of *Streptomyces* species, but also at a previously uncharacterized *attB*-like locus in SCO3793. Transcriptome profiling at the 24^th^ and 72^nd^ hours of fermentation revealed extensive Lon-dependent remodeling, particularly in key functional categories such as secondary metabolism, stress response, primary metabolism, and morphological differentiation. Confocal microscopy confirmed that *lon* overexpression shifts programmed cell death dynamics, triggers earlier MII (antibiotic-producing mycelia) formation, and supports sustained viability and homogeneous pellet morphology, even in the later stages of fermentation. These structural features coincide with elevated antibiotic titers. Together, these results position Lon as a systems-level regulator, which couples proteostasis to metabolic flux, nutrient signaling, developmental progression, and secondary metabolism. By mapping Lon-dependent regulatory networks, linking transcriptional signatures to quantitative morphological phenotypes, and identifying a new chromosomal *attB*-like integration site, this work provides a valuable framework for protease-guided strain engineering and highlights Lon as a promising lever for rational improvement of antibiotic production in *Streptomyces*. To the best of our knowledge, this is the first comprehensive study investigating the systems-level effects of overexpression of Lon protease gene in *Streptomyces*.

## 1 Introduction

*Streptomyces* species are notable producers of bioactive natural products, including many frontline antibiotics, antifungals, immunosuppressants, and antitumor agents (Barka et al., 2016). Approximately two-thirds of the currently approved antibiotics originate from actinomycetes, and the majority of these (around 80%) are derived from *Streptomyces*, whose genomes contain an expansive repertoire of cryptic biosynthetic gene clusters (Alam et al., 2022; Donald et al., 2022). The biosynthesis of secondary metabolites is known to be triggered by environmental stress signals and controlled by intricate molecular networks involving multiple families of regulatory proteins (Bibb, 2005).

In *Streptomyces*, the synthesis of secondary metabolites is closely linked to its complex life cycle (Manteca and Yagüe, 2018). Development in both solid and submerged cultures proceeds through two distinct mycelium stages: primary mycelium (MI, compartmentalized mycelia) and secondary mycelium (MII, antibiotic-producing mycelia). Growth begins with the formation of MI following spore germination. MI cells subsequently undergo highly ordered programmed cell death (PCD). The remaining viable segments then differentiate into the MII which are multinucleated, antibiotic-producing mycelia (Manteca et al., 2008; Yagüe et al., 2014; Manteca and Yagüe, 2018). This PCD-driven morphological remodeling results in pellet and clump formation in submerged cultures and aerial mycelium formation and sporulation in solid cultures. Together these processes shape the organism's characteristic growth phases (Yagüe et al., 2010, 2014).

The remarkable biosynthetic versatility of *Streptomyces* is achieved through multilayered regulatory networks that coordinate different processes, including morphological differentiation, metabolic fluxes, and stress responses (Manteca et al., 2008; Van Wezel and McDowall, 2011; Liu et al., 2013; Manteca and Yagüe, 2018). ATP-dependent Lon protease is a central node in proteostasis and provides a strategic leverage point for rewiring the cell's transcriptional network. Lon is an evolutionarily conserved AAA^+^ protease that plays a key role in cellular physiology by degrading misfolded or damaged proteins, as well as numerous regulatory proteins (Tsilibaris et al., 2006; Lee and Suzuki, 2008; Gur et al., 2011). As a pleiotropic regulator in bacteria, Lon governs various developmental processes, including sporulation (Tojo et al., 1993; Liu et al., 1999), the cell cycle (Wright et al., 1996; Omnus et al., 2021) and capsule synthesis (Torres-Cabassa and Gottesman, 1987). It also governs behavioral processes, such as quorum sensing (Takaya et al., 2008), biofilm formation and motility (Marr et al., 2007), and pathogenicity (Kirthika et al., 2023). Lon is also required for adaptation to diverse environmental stress conditions, including the UV stress (Mizusawa and Gottesman, 1983; Barkad et al., 2021; Yilmaz et al., 2025) and the SOS response (Breidenstein et al., 2012), nutrient starvation (Kuroda, 2006), and osmotic stress (Barkad et al., 2021; Yilmaz et al., 2025).

Moreover, studies have shown that Lon can influence the biosynthesis of bioactive compounds; for instance, it represses pyoluteorin biosynthesis in *Pseudomonas fluorescens* Pf-5 (Whistler et al., 2000), whereas its overexpression has been reported to increase endotoxin production in *Bacillus thuringiensis* (Barkad et al., 2021). The role of the Lon protease in *Streptomyces* species has been investigated only to a limited extent; Sobczyk et al. demonstrated that *lon* is part of the HspR/HAIR heat-shock response regulon, yet its impact on secondary metabolism has not been examined in this study (Sobczyk et al., 2002). Previously, we demonstrated that recombinant strains of *Streptomyces coelicolor* A3(2) overexpressing the *lon* gene exhibited a significant increase in the production of actinorhodin and undecylprodigiosin (Demir et al., 2019; Yilmaz et al., 2025). The molecular mechanisms, by which Lon reshapes transcription networks, primary metabolism, and developmental progression, influencing secondary metabolism and resulting in high antibiotic production in *S. coelicolor* are unknown. In this study, we address this knowledge gap by integrating whole-genome sequencing (WGS), transcriptome profiling, and advanced imaging to dissect Lon-mediated regulation in *S. coelicolor* A3(2).

## 2 Materials and methods

### 2.1 Microorganisms and culture conditions

For this study, we used the wild-type (WT) strain of *S. coelicolor* A3(2) (Hopwood, 1999), as well as its recombinant strains. The recombinant strains, designated as Sco-pRA and Sco-pRA*lon*, were previously generated through the insertion of the integrative pRA plasmid with apramycin resistance gene and the pRA*lon* plasmid with *lon* protease gene into the *S. coelicolor* A3(2) genome, respectively (Demir et al., 2019). pRA and pRA*lon* are ϕC31-based integrative vectors. The *Streptomyces* strains were cultivated at 30 °C in TBO for sporulation, TSB and R2YE for fermentation, and YEME media for DNA isolation and scanning electron microscopy (SEM) analysis. The media were supplemented with apramycin (50 μg/ml) when necessary.

### 2.2 DNA isolation, whole genome sequencing and bioinformatics analysis

A volume of 200 μl spores of Sco-pRA and Sco-pRA*lon* strains were grown in YEME medium at 30 °C for 2 days. An aliquot of this pre-culture was then inoculated into fresh YEME medium at a ratio of 1:10 (v/v) and incubated under the same conditions. After incubation, genomic DNA was isolated using the NucleoSpin Microbial DNA Isolation Kit (Macherey-Nagel, Germany) according to manufacturer's instructions. The concentration and purity of the final DNA product were determined by spectrophotometric measurements using a NanoDrop (BioDrop, UK). The isolates were then sent to BGI (Hong Kong) for WGS. A bioinformatics analysis of the WGS raw data was performed using the FASTQC (Andrews, 2010), Trimmomatic (Bolger et al., 2014), Burrows-Wheeler Aligner (Li and Durbin, 2009), and Samtools (Li et al., 2009) programs. The “Genome Analysis Toolkit” (McKenna et al., 2010) was used in the variant acquisition processes, while the “Ensembl Variant Effect Predictor (VEP)” (McLaren et al., 2016) was employed in the variant annotation processes. The break and insertion regions were determined using custom-written R Studio codes (Team, 2021).

### 2.3 RNA isolation

For RNA isolation, 200 μl of *S. coelicolor* A3(2), Sco-pRA, and Sco-pRA*lon* spores were grown separately in TSB medium at 30 °C for 2 days. Equal wet-weight cell pellets were harvested from the pre-cultures and inoculated into R2YE medium. Samples were collected from the cultures at 24, 36, 48, 60, 72, and 96 h after inoculation. The cell pellets were dissolved in 200 μl of TE buffer and approximately 70 μl of lysozyme (10 mg/ml) was added. The mixture was then incubated at 37 °C for 60 min. After the preliminary lysis step, total RNA was isolated using a NucleoSpin RNA Plus isolation kit (Macherey-Nagel GmbH & Co, Germany), according to the manufacturer's instructions. To ensure the purity of the RNA samples, deoxyribonuclease I (Thermo Scientific, USA) was used to eliminate any possible DNA contamination. The concentration and purity of the RNA were determined by spectrophotometric measurements using a NanoDrop (BioDrop, UK), and the RNA integrity value (RIN) was determined using an Agilent 2100 Bioanalyzer (USA). After quantification, the RNA was divided into aliquots and stored at −80 °C for future use.

### 2.4 Library construction, RNA sequencing, and bioinformatic analysis

The RNA samples from the *S. coelicolor* A3(2), Sco-PRA, and Sco-pRA*lon* strains were sequenced using a 2 × 150 bp paired-end protocol on the Illumina HiSeq 2000 platform (BGI, Hong Kong). Sequencing was performed with two biological replicates of each strain. The adapter sequences, unknown bases, residual rRNA sequences, and low-quality reads were filtered out using the SOAPnuke v1.5.2 internal software (Chen et al., 2018). The clean reads obtained after filtering were stored in FASTQ format and mapped using HISAT2 (Kim et al., 2015). For gene expression analysis, the clean reads were aligned to the *S. coelicolo*r reference genome (GenBank accession number: AL645882.2) using Bowtie2 (Langmead and Salzberg, 2012). Gene expression levels were then calculated using RSEM (Li and Dewey, 2011) and normalized to obtain fragments per kilobase of transcript per million mapped reads (FPKM) values for each gene. The differentially expressed genes (DEGs) between the WT and ScoPRA*lon* were identified with DESeq2 (Love et al., 2014). Transcripts showing at least two-fold change in expression level (|log_2_(FoldChange)| ≥ 1), a *P*-value ≤ 0.05, and a false discovery rate (FDR) value ≤ 0.05 were identified as significantly differentially expressed genes (Benjamini and Yekutieli, 2001; Supplementary Tables S1, S3). Gene ontology (GO) enrichment and Kyoto Encyclopedia of Genes and Genomes (KEGG) pathway enrichment analyses were performed using Hyper, an R Studio function, to categorize the DEGs. The raw sequencing data were deposited on the NCBI SRA website (http://www.ncbi.nlm.nih.gov/sra/) under the Bioproject accession number; PRJNA1077142.

### 2.5 Quantitative real-time PCR (RT-qPCR)

RNA samples were analyzed by RT-qPCR to determine which subset should undergo further sequencing. The results were also used to compare and validate the RNA-seq analysis results. The primers used were designed through web-based tools (Primer 3, Primer Blast and OligoAnalyzer Tool-DT) and are presented in Supplementary Table S2. The cDNA synthesis was performed using 300 ng of RNA and random hexamer primers with the iScript cDNA Synthesis Kit (Bio-rad, USA). RT-qPCR reactions were performed using SYBR Green qPCR Master Mix (Bio-rad, USA) in a StepOnePlus™ Real-Time PCR System (Applied Biosystems). The reaction components were as follows: 10 μl SYBR Green supermix (2X), 3 μl cDNA (30 ng), 0.8 μl (10 μM) F primer, 0.8 μl (10 μM) R primer and 5.4 μl dH_2_O to give a final volume of 20 μl. The RT-qPCR thermal profile included an initial denaturation step (10 min at 95 °C) followed by 40 cycles of amplification (15 min at 95 °C and 1 min at 60 °C). Melting curve analysis was performed at a range of 55–95 °C in 0.1 °C increments to evaluate the specificity of the primers. The reference genes *gyrA* and *hrdB* were used for the normalization of the gene expression, while cDNAs from the *S. coelicolor* A3(2) strain were used as positive controls. Changes in relative gene expression values were calculated using the formula 2^−ΔΔCT^ (Livak and Schmittgen, 2001). Statistical analyses of gene expression levels were performed using the Mann-Whitney test in GraphPad Prism 10.6.1 software.

### 2.6 Confocal laser scanning microscopy (CLSM) analysis

The LIVE/DEAD Bac-Light Bacterial Viability Kit (L-7012) was used for the confocal analysis (Fernandez and Sanchez, 2001). The samples were prepared by cultivating *Streptomyces* cells in R2YE medium at 30 °C and at 200 rpm. Cultures of 1 ml were collected at 24-h intervals during the 5-day fermentation process. The pellets obtained by centrifugation of the samples at 12.000 rpm for 5 min were washed twice with 1 ml of dH_2_0. According to the kit instructions, the SYTO 9 and PI nucleic acid staining mixture was subsequently added to the pellets. After a 10 min incubation at room temperature in the dark, 20 μl of the mixture was spread onto a clean microscope slide. The prepared slides were then imaged using a Zeiss LSM 880 confocal microscope at 488 nm and 568 nm excitation and 530 nm (green) or 630 nm (red) emission wavelengths.

### 2.7 Scanning electron microscopy (SEM) analysis

For the SEM analysis, the sterilized aluminum stubs were inserted at an angle of about 45° into YEME agar plates until approximately half of the stub was dipped into the medium. Bacterial samples were then spread along the line where the surface of the stubs met the medium by using an inoculating loop. After 12 days of incubation at 30 °C, the upper surface of each stub was then coated with gold and examined under the scanning electron microscope (Philips XL 30 SFEG; Kumar et al., 2011).

## 3 Results

### 3.1 Results of WGS analysis for identifying the location(s) where the pRA*lon* and pRA plasmids integrates into the genome

In a previous study, the *lon* gene, together with its native promoter, was successfully cloned into the integrative vector pRA which has ϕC31 *int*/*attP* site (Demir et al., 2019). Subsequently, the recombinant plasmid was integrated into the *S. coelicolor* A3(2) genome. Remarkably, the recombinant strain, SCO-pRA*lon*, exhibited a notable increase in antibiotic production compared to the wild type (Demir et al., 2019). Through WGS coupled with bioinformatic analysis revealed that pRA*lon*, which has a ϕC31 *int*/*attP* site, was integrated into two distinct regions in the genome, separated by 7 kilobases. As anticipated, the first integration site was identified within the *SCAC2.06c* gene (SCO3798), which encodes a putative chromosome condensation protein (Supplementary Figure S1). It has been established that the integration of integrative vectors into this specific *attB* region is 300 times more effective (Combes et al., 2002). The second integration region was identified within the *SCAC2.01* (SCO3793) gene, which encodes a hypothetical protein. This region is entirely novel and distinct from the pseudo-*attB* regions identified to date (Combes et al., 2002; Supplementary Figure S1). Since each integrated pRA*lon* construct carries a single *lon* gene, the Sco-pRA*lon* strain harbors a total of three *lon* gene copies in its genome (one native chromosomal copy and two integrated copies). To be used as the control strain, the empty plasmid pRA was also integrated into the *S. coelicolor* genome resulting in Sco-pRA. According to the WGS results, pRA was found to be integrated into a single location: the *attB* region within the SCAC2.06c (SCO3798) gene, as expected (Supplementary Figure S2).

### 3.2 General features of the RNA-seq data: recombinant Sco-pRA*lon* strain vs. the wild type

We isolated total RNA from the *S. coelicolor* A3(2) wild type (Sco), Sco-pRA and Sco-pRA*lon* strains at the 24^th^ and 72^nd^ hours of fermentation, then performed sequencing with two biological replicates. We decided to sequence the samples from the 24^th^ and 72^nd^ hours after conducting a preliminary study (Supplementary Figure S3). A significant difference in *lon* gene expression was observed in the Sco-pRA*lon* strain compared to the wild type Sco strain at the 24^th^ hour of fermentation. The 72^nd^ hour was the time point at which *lon* and *actII-ORF4*, which encodes the actinorhodin activator protein, exhibited the highest expression levels (Supplementary Figure S3).

Following RNA sequencing, we conducted a comparative analysis of the expression levels of all identified transcripts. For clarity, this section only compares data from the recombinant Sco-pRA*lon* strain and the wild-type strain.

**Figure 1:**
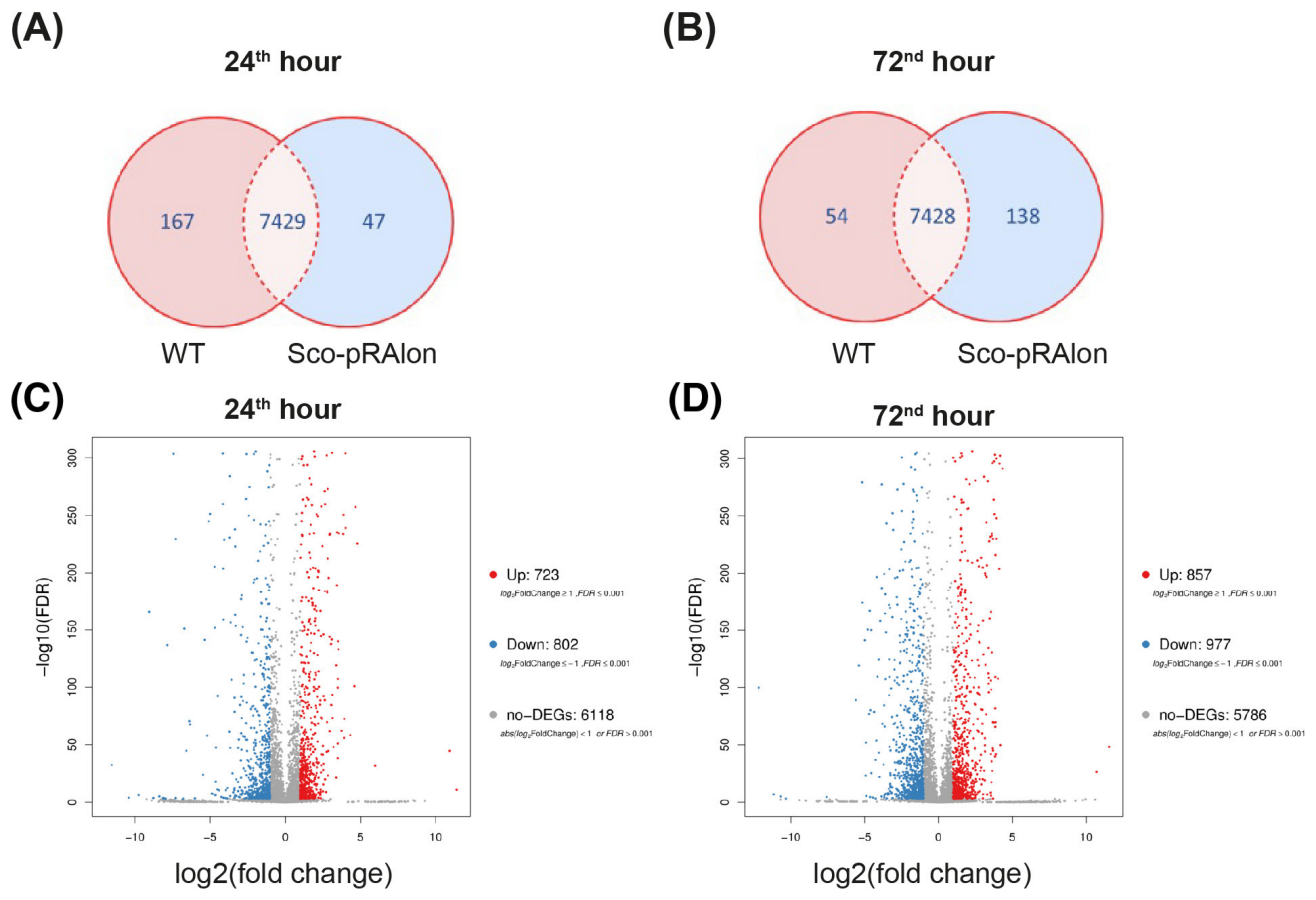
Overview of the RNA-Seq analysis. **(A)** Venn diagram showing the distribution of genes commonly or exclusively expressed in the Sco-pRA*lon* and WT strains at 24^th^ hour and **(B)** 72^nd^ hour. **(C)** A volcano plot illustrating the differentially expressed genes (DEGs) between the Sco-pRA*lon* strain and WT at 24^th^ hour and **(D)** 72^nd^ hour. The *X*-axis represents the value of log_2_ (fold change, FC) and, the *Y*-axis represents the value of –log10 (false discovery rate, FDR). Genes with a log_2_ fold change ≥ 1 and an adjusted p-value ≤ 0.001 (red dots) are considered significantly upregulated, and genes with a log_2_ fold change ≤ −1 and an adjusted *p*-value ≤ 0.001 (blue dots) are considered significantly downregulated. Gray dots indicate non-significant genes with an absolute log_2_ fold change value of <1.

After filtering the raw data, an average of 35.68 Mb of clean reads were obtained per sample. These reads mapped to the reference genome with an average ratio of 95.01%, and a total of 7,729 genes were successfully detected. Further analysis revealed that a total of 7,429 genes were expressed in both strains, while 167 and 47 genes were only expressed in the WT and Sco-pRA*lon* strains, respectively, at the 24^th^ hour (Figure 1A). At the 72^nd^ hour, we detected 7,428 genes that were commonly expressed in both strains. The number of genes exclusively expressed in the WT strain decreased to 54, while the number of genes expressed in the Sco-pRA*lon* strain increased to 138 (Figure 1B). Subsequent gene expression analyses revealed the total number of upregulated and downregulated DEGs in the Sco-pRA*lon* strain and WT strain, as depicted in the volcano plot graphs (Figure 1). Specifically, 1,525 genes exhibited differential expression at the 24^th^ hour, with 723 up-regulated and 802 down-regulated genes (Figure 1C). By the 72 h, the number of DEGs had increased to 1,834, including 857 up-regulated and 977 down-regulated genes (Figure 1D). These findings underscore a heightened transcriptional activity in the recombinant strain at the 72^nd^ hour compared to the 24^th^ hour.

### 3.3 GO analysis of DEGs

**Figure 2:**
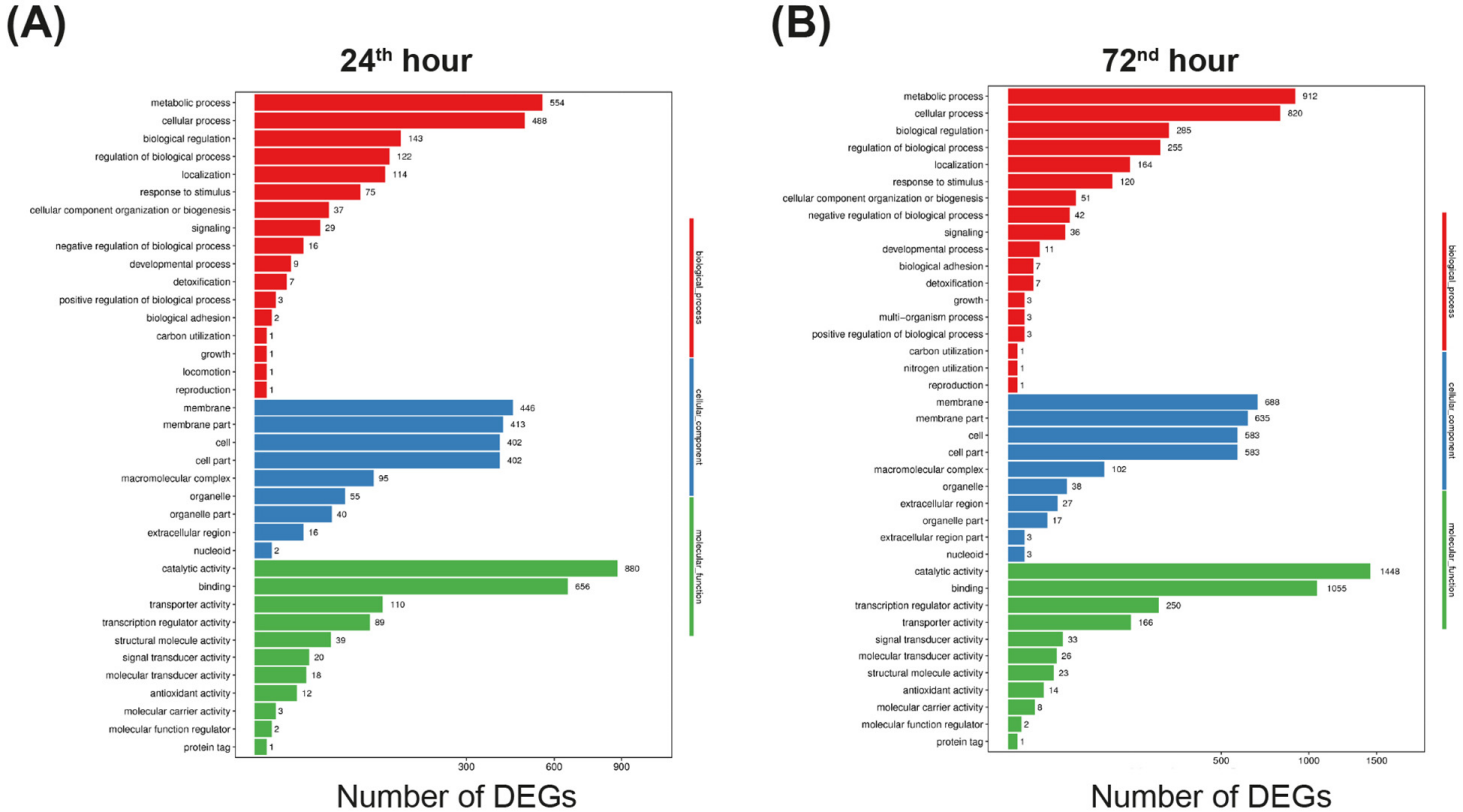
GO functional classification map of significant DEGs at 24^th^ hour **(A)** and at 72^nd^ hour, color coded based on the main GO domain **(B)**. *X* axis represents number of DEGs. *Y* axis represents the GO term.

GO classification and functional enrichment analyses were performed to determine the functions of the DEGs identified by the comparisons between the SCO-pRA*lon* and wild-type cDNA libraries. The DEGs were categorized into three main GO domains: biological process, molecular function, and cellular component. At the 24^th^ and 72^nd^ hours of fermentation, 37 and 39 significantly enriched and highly similar GO terms were identified, respectively (Figure 2). The highest number of DEGs were found in the categories of catalytic activity, binding, metabolic processes, cellular processes, and membrane components at both time points. Notably, the number of DEGs identified at the 72^nd^ hour time point was higher in almost all categories (Figure 2B).

### 3.4 KEGG pathway analysis of DEGs

**Figure 3:**
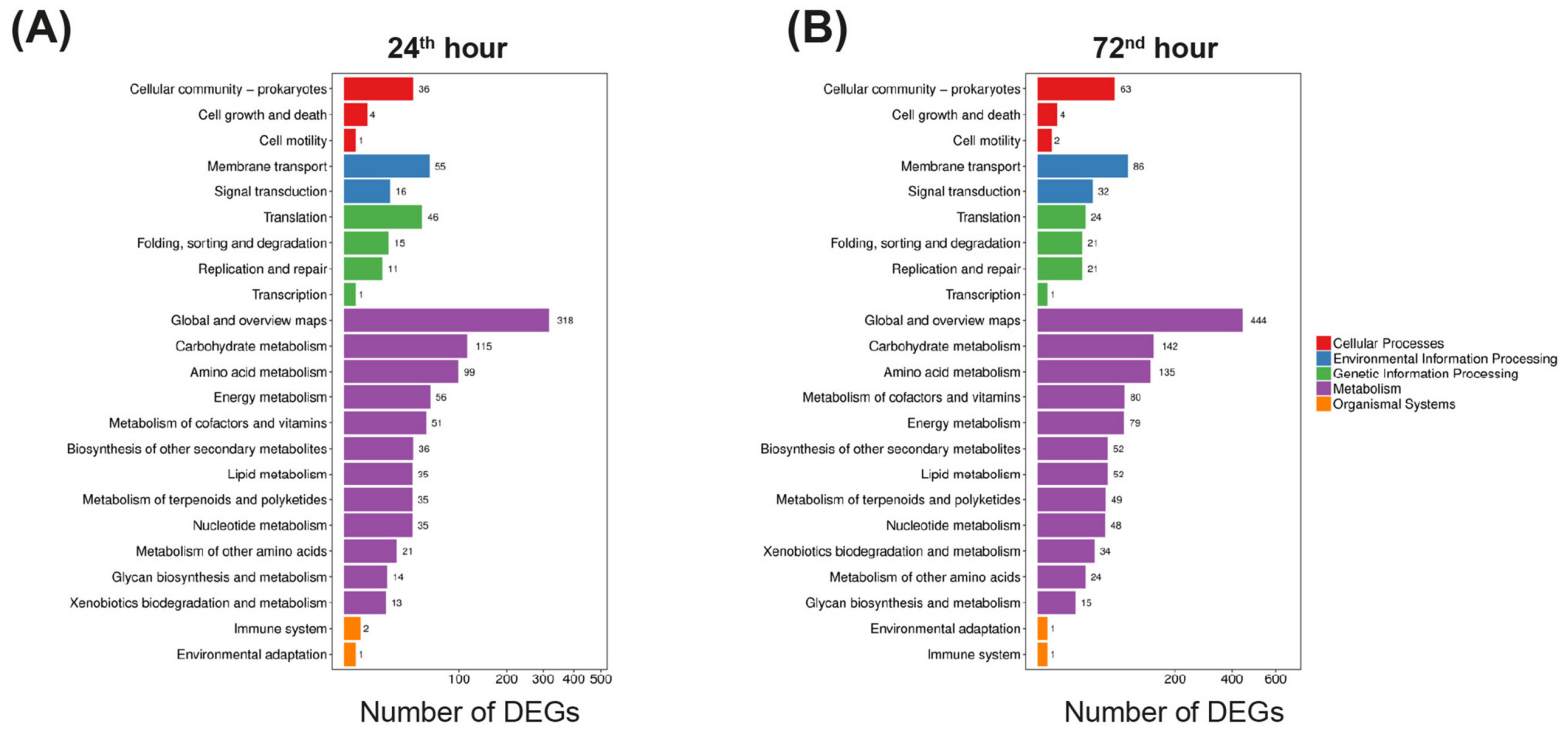
The KEGG pathway classification maps of the significant DEGs at the 24^th^ hour **(A)** and 72^nd^ hour **(B)** are color coded by the KEGG pathway categories. The *X*-axis represents the number of DEGs, and the *Y*-axis represents the pathway name.

KEGG pathway analysis revealed significant differences in the biological pathways associated with the DEGs in the high-antibiotic-producing strain Sco*-*pRA*lon*. Based on the KEGG pathway classification, 1,016 and 1,410 DEGs, found at 24^th^ and 72^nd^ hour timepoints, respectively, were assigned to 5 categories: cellular processes, environmental information processing, genetic information processing, metabolism and organismal systems. At both time points, the DEG ratios of the individual categories were very similar, with the metabolism pathway having the highest ratio of about 83%. Approximately 9% of the total DEG fraction is related to terpenoid and polyketide metabolism and the biosynthesis of other secondary metabolites in this pathway (Figure 3).

**Figure 4:**
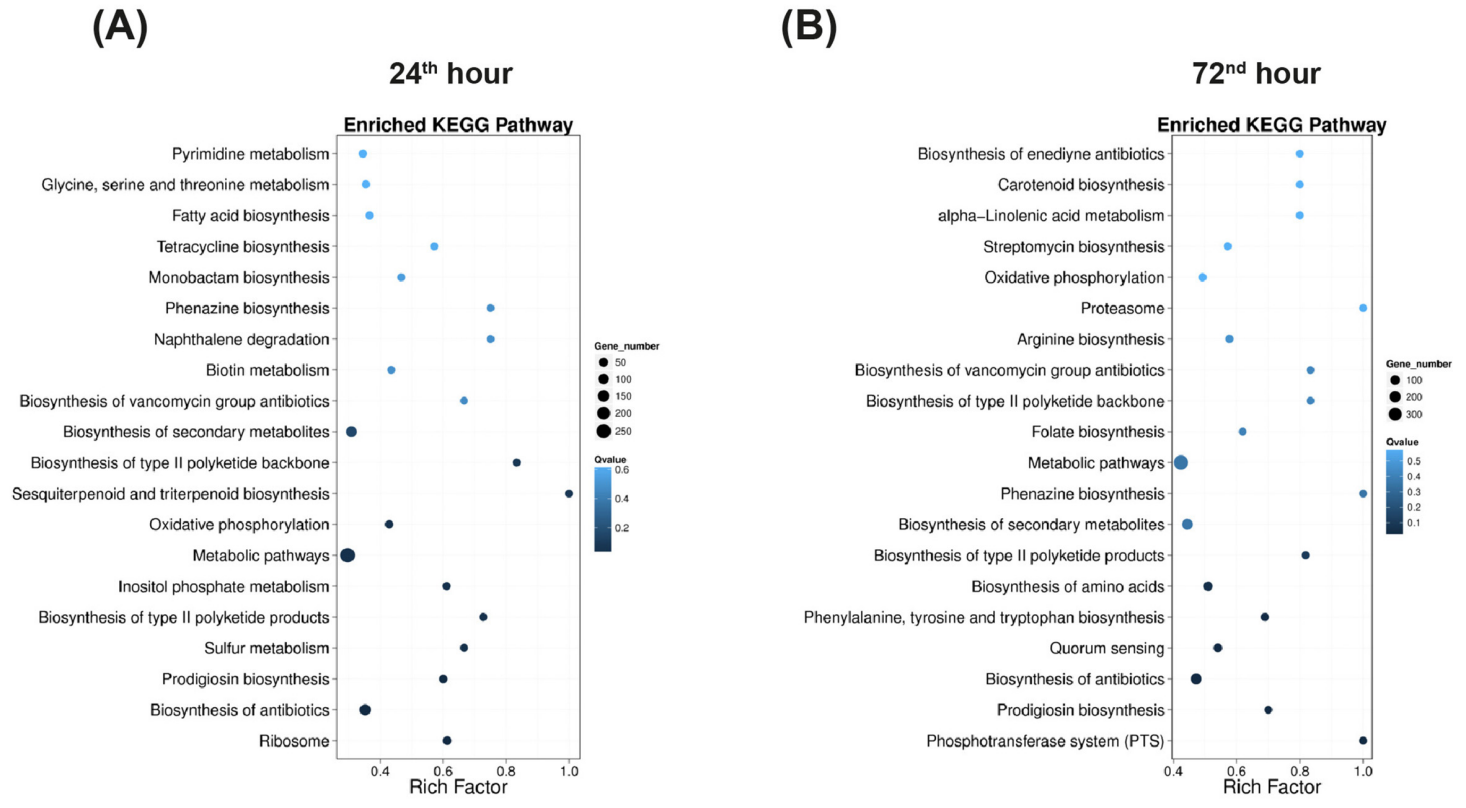
Bubble diagram of the functionally enriched KEGG pathways of DEGs at 24^th^ hour **(A)** and 72^nd^ hour **(B)**. The *X*-axis indicates the enrichment factor, and the *Y*-axis indicates the pathway name. Bubble size represents the number of genes belonging to a specific KEGG pathway. Color represents the enriched *Q* value, with lighter colors indicating a higher *Q* value. The color bar is shown on the right.

Next, KEGG pathway functional enrichment analysis was used to determine the distribution of DEGs in the recombinant strain more specifically. Metabolic pathways and pathways related to antibiotic and other secondary metabolite biosynthesis were among the top 20 enriched pathways with the highest DEG numbers for both time points (Figure 4). Supplementary Figure S4 shows the distribution of up- and down-regulated DEGs belonging to the 30 significantly enriched pathways in the SCO-PRA*lon* strain. At the 24^th^ hour, there was a trend toward increased expression levels of genes involved in secondary metabolic pathways, including the biosynthesis of some antibiotics. Conversely, the expression levels of genes related to the biosynthesis of amino acids such as glycine, serine, threonine, valine, and leucine, which are indicative of primary metabolism, tend to decrease (Supplementary Figure S4A). At the 72^nd^ hour, differences in the ratio of up- and down-regulated DEGs were observed; however, a similar alteration was seen for both primary and secondary metabolisms (Supplementary Figure S4B). Although gene expression is not necessarily directly proportional to the amount of antibiotic produced, it is clear that the recombinant strain shifts carbon flux from primary to secondary metabolism. The following sections further elucidate these metabolic switches by examining the expression levels of important DEGs and their physiological functions in *S. coelicolor* A3(2).

### 3.5 Categorization of DEGs in the Sco-pRA*lon* strain

**Figure 5:**
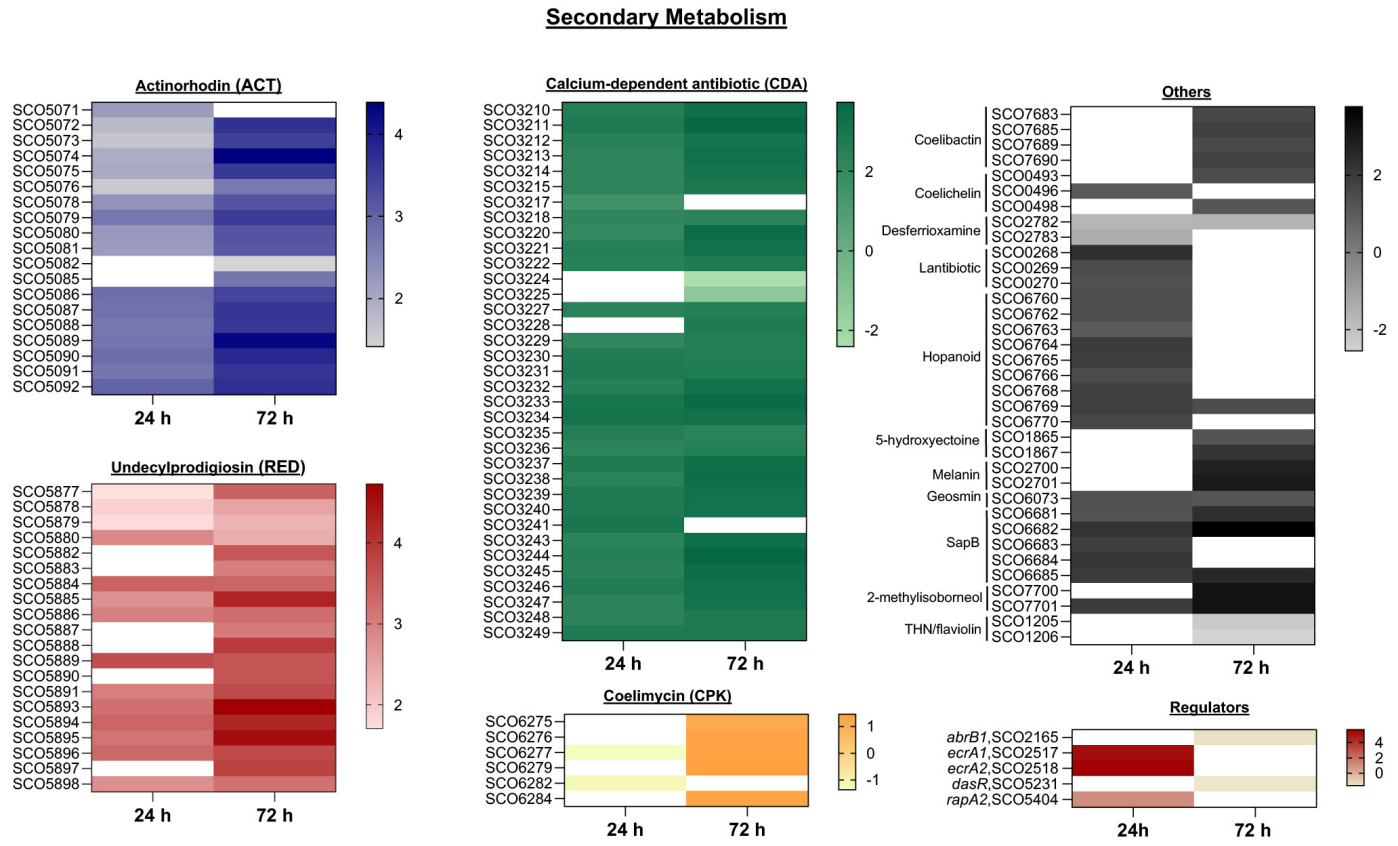
Heat map showing the DEGs involved in secondary metabolite biosynthesis between the *S. coelicolor* A3(2) wild-type strain and the Sco-pRA*lon* recombinant strain. Genes with a |log_2_ (fold change)| ≥ 1 and *P* ≤ 0.05 with FDR ≤ 0.05 were considered to be significantly differentially expressed. RNA samples were prepared from cultures of the strains grown in R2YE liquid medium for 24^th^ and 72^nd^ hours. For exact values see Supplementary Table S3.

**Figure 6:**
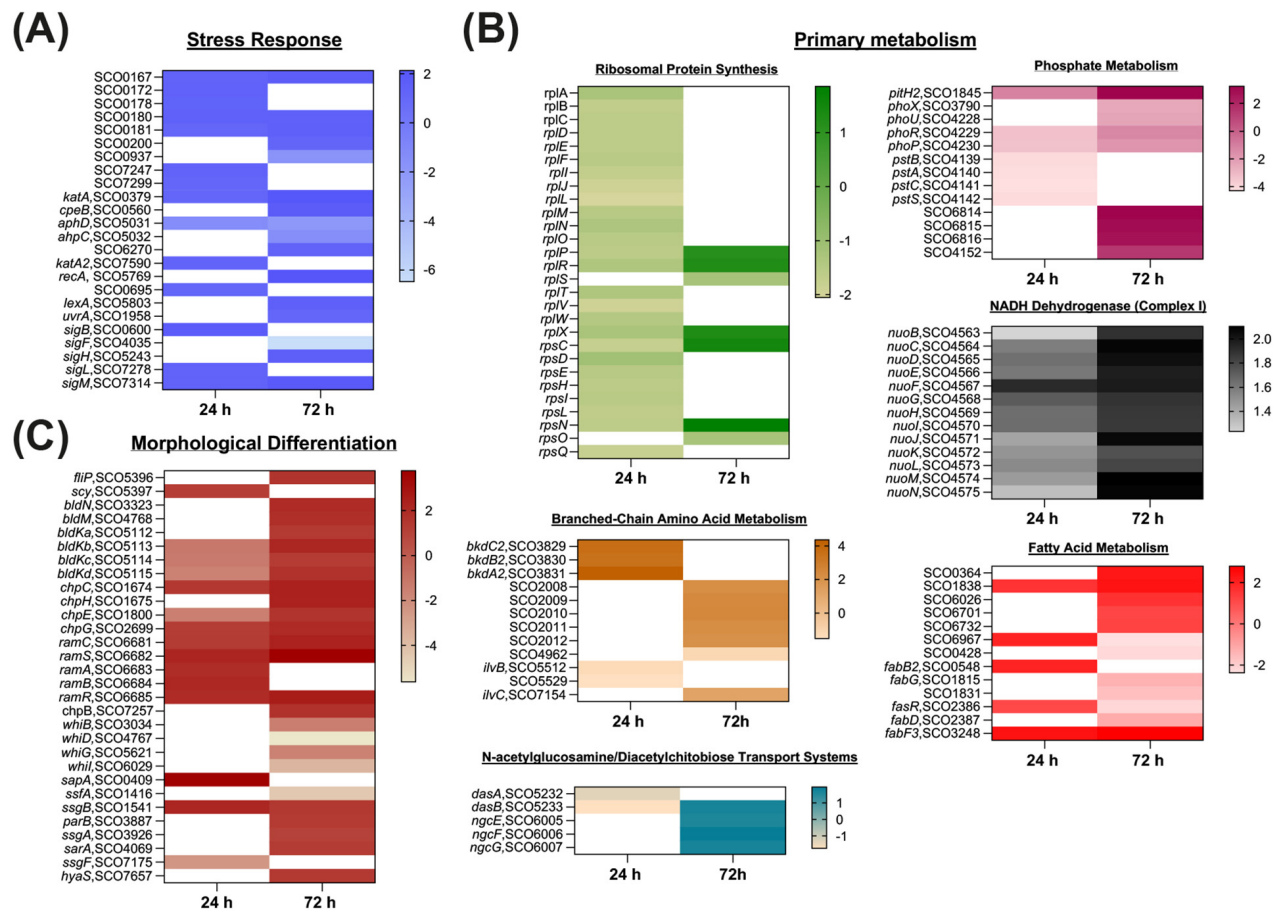
Heat maps showing the DEGs involved in **(A)** stress response, **(B)** primary metabolism, and **(C)** morphological differentiation between *S. coelicolor* A3(2) wild-type and Sco-pRA*lon* recombinant strains. Genes with a |log_2_ (fold change)| ≥ 1 and *P* ≤ 0.05 with FDR ≤ 0.05, were considered to be significantly differentially expressed. RNA samples were prepared from cultures of the strains grown in R2YE liquid medium for 24^th^ and 72^nd^ hours. For exact values see Supplementary Table S3.

RNA-seq analysis revealed that the high antibiotic-producing recombinant strain had DEGs involved in four main biological processes: secondary metabolism, stress response, primary metabolism and morphological differentiation (Figures 5, 6, Supplementary Table S3).

#### 3.5.1 Secondary metabolism

**Figure 7:**
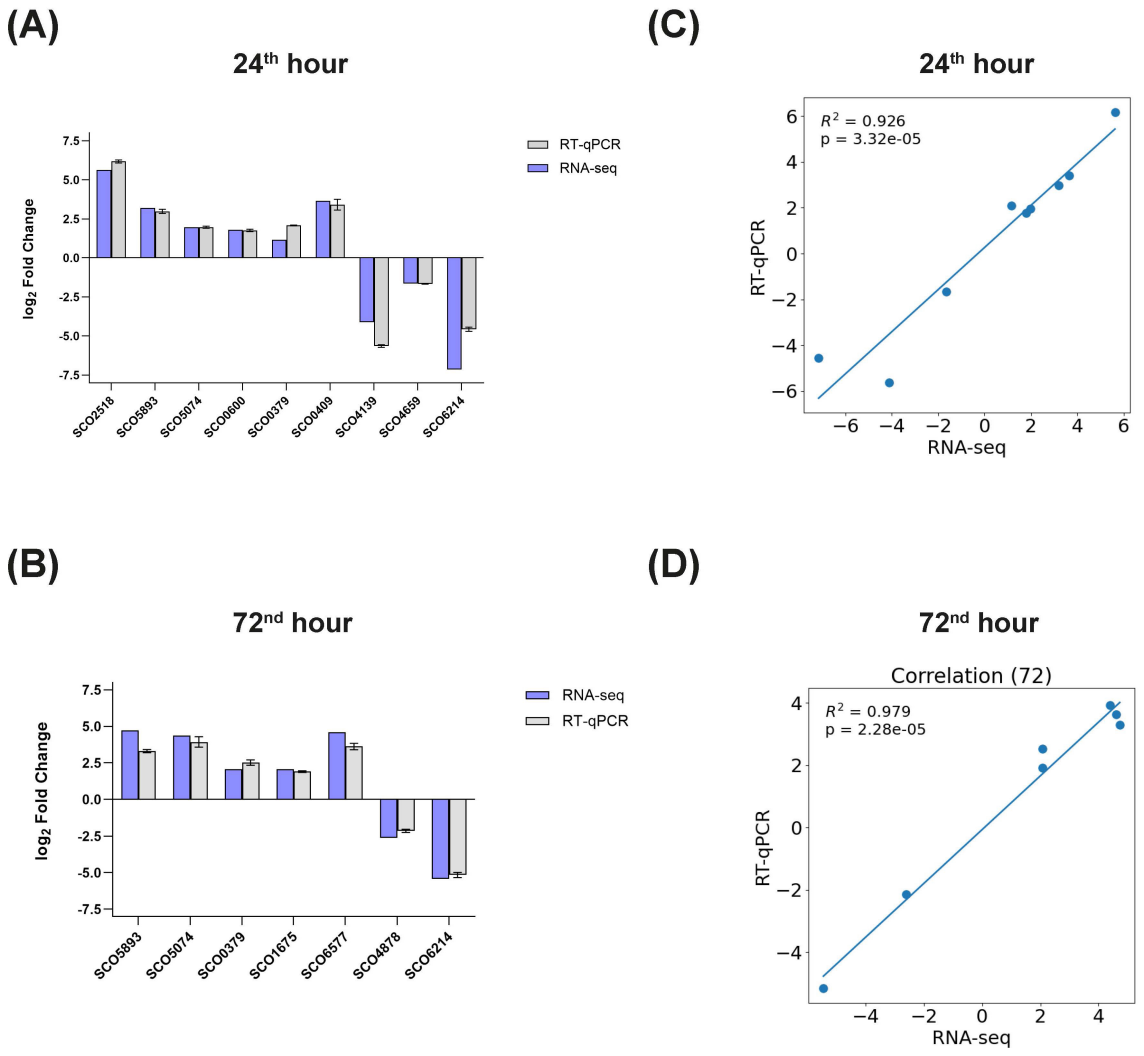
Validation of the RNA-seq measurements. **(A)** Comparison of log_2_ fold changes in the expression levels of several Sco-pRAlon and WT genes, as measured by RNA-seq (blue bars) and RT-qPCR (gray bars) at the 24^th^ and **(B)** 72^nd^ hours of fermentation. Error bars represent standard deviation from the mean. The significance threshold for the analysis of differentially expressed genes a *p*-value of < 0.05 for the RNA seq and RT-qPCR experiments. **(C)** Correlation between RNA-seq and RT-qPCR-based gene expression analysis at 24^th^ hour and **(D)** 72^nd^ hour. The linear regression line and Pearson correlation coefficient are indicated in the figures.

Approximately 30 biosynthetic gene clusters responsible for metabolite production have been identified in the fully sequenced *S. coelicolor* genome (Bentley et al., 2002; Challis and Hopwood, 2003; Jeong et al., 2016). In the Sco-pRA*lon*, significant expression changes were observed in multiple gene clusters, with upregulation being predominant (Figure 5, Supplementary Table S3). Antibiotic biosynthetic clusters, including ACT (SCO5071–SCO5092), the RED (SCO5877–SCO5898), and the calcium-dependent antibiotic (CDA; SCO3210–SCO3249), showed strong induction. Expression levels increased up to 13-fold at 24^th^ hour and up to 27-fold at 72^nd^ hour. Pathway-specific regulatory genes were also upregulated: *actII-ORF4* (~6.6-fold at 72^nd^ hour), *redD* (~3.3-fold at 24^th^ hour and ~10.6-fold at 72^nd^ hour), and *cdaR* (~2.9-fold at 24^th^ hour). In addition, six differentially expressed genes were identified within the coelimycin (CPK) biosynthetic cluster (SCO6273–SCO6288; Figure 5, Supplementary Table S3). RT-qPCR validation of *redK* (SCO5893) and *actVI-3* (SCO5074) showed strong concordance with the RNA-seq data at both time points (Figure 7).

Genes associated with siderophore biosynthesis displayed cluster-specific expression patterns. Coelibactin cluster genes (SCO7683, SCO7685, SCO7689, and SCO7690) exhibited 3.5-fold upregulation at 72^nd^ hour, while coelichelin cluster genes (SCO0493, SCO0496, and SCO0498) showed ~2.8-fold increase at the same time point. In contrast, desferrioxamine biosynthetic genes SCO2782 and SCO2783 were downregulated (~3.2-fold and ~2.7-fold, respectively; Figure 5, Supplementary Table S3).

Additional biosynthetic clusters exhibited positive regulation at both time points, including those involved in lantibiotic (SCO0267–SCO0270), hopanoid (SCO6759-SCO6771), 5-hydroxyectoine (SCO1864–SCO1867), melanin (SCO2700–SCO2701), geosmin (SCO6073), SapB (SCO6681–SCO6685), and 2-methylisoborneol (SCO7700–SCO7701) biosynthesis. In contrast, THN/flaviolin biosynthesis genes SCO1205 and SCO1206 showed a ~4.8-fold decrease in expression at 72^nd^ hour (Figure 5, Supplementary Table S3).

Significant changes in the expression of several TCS-related genes were observed in the recombinant strain. The response regulator *abrB1* (SCO2165) was downregulated by approximately 2.9-fold at 72^nd^ hour, whereas *rapA2* (SCO5404) was upregulated (~3-fold at 24^th^ hour). *ecrA1* (SCO2517) and *ecrA2* (SCO2518) were strongly upregulated (~36.4–49.2-fold) at 24^th^ hour, this result was confirmed for *ecrA2* by RT-qPCR. In contrast, the global regulator *dasR* (SCO5231; Świa̧tek-Połatyńska et al., 2015) was downregulated by ~3.2-fold at 72^nd^ hour (Figure 5, Supplementary Table S3).

#### 3.5.2 Stress response

Significant transcriptional changes were detected in genes involved in various stress responses in the Sco-pRA*lon* strain compared to the wild type strain (Figure 6A, Supplementary Table S3). Notably, genes encoding universal stress proteins (SCO0167, SCO0172, SCO0178, SCO0180, SCO0181, SCO0200, SCO7247 and SCO7299) were found to be up-regulated by up to 2.75-fold at 24^th^ hour and up to 3.14-fold at 72^nd^ hour of fermentation. In contrast, only SCO0937 exhibited downregulation, showing an approximately 3.5-fold decrease at 72^nd^ hour.

Genes involved in the oxidative stress response also showed differential expression. Catalase encoding genes *katA* (SCO0379), *cpeB* (SCO0560), *katA2* (SCO7590) and SCO6270 exhibited a consistent trend of positive regulation. Conversely, the expression of *aphD* (SCO5031) and *aphC* (SCO5032), which exhibit oxidoreductase activity, showed negative regulation.

Marked induction was also observed in SOS response–related genes. The expression levels of *uvrA* (SCO1958), *lexA* (SCO5803) and *recA* (SCO5769) increased markedly (up to 4.4 fold) particularly at 72^nd^ hour. In addition, SCO0695, which encodes a conserved hypothetical protein, exhibited a 2.23-fold upregulation at 24^th^ hour.

Sigma factors associated with the general stress response (Kormanec et al., 2016) displayed notable expression changes in the recombinant strain. *sigB* (SCO0600) and its associated factors *sigH* (SCO5243), *sigM* (SCO7314), and *sigL* (SCO7278) were upregulated by up to 3.5 fold at 24^th^ hour and up to 3.7-fold at 72^nd^ hour. In contrast, *sigF* (SCO4035), involved in the late stages of sporulation (Potúčková et al., 1995), was strongly down-regulated (~89-fold) at 72^nd^ hour. RT-qPCR validation confirmed the positive regulatory trend observed in *katA* (SCO0379) and *sigB* (SCO0600; Figure 7).

#### 3.5.3 Primary metabolism

##### 3.5.3.1 Ribosomal protein synthesis

The majority of genes encoding both the large (*rpl*) and small (*rps*) ribosomal subunits were markedly down-regulated in the Sco-pRA*lon* strain compared to the wild-type at 24^th^ hour of fermentation (e.g., *rplB, rplC, rplD, rplF, rplJ, rplM; rpsE, rpsH, rpsI, rpsL*). At 72^nd^ hour, although fewer DEGs were associated with ribosomal subunits, several genes (*rplP, rplR, rplX, rpsN*) exhibited a low-level upregulation trend (Figure 6B; Supplementary Table S3). RT-qPCR analysis confirmed the ~3.12-fold downregulation of *rpsL* (SCO4659) at 24^th^ hour, which is consistent with the RNA-seq data (Figure 7).

##### 3.5.3.2 Branched-chain amino acid metabolism

The *S. coelicolor* genome contains two distinct BCDH (branched-chain n-keto acid dehydrogenase) gene clusters that are involved in catabolizing branched-chain amino acids (valine, isoleucine, and leucine), namely *bkdA1B1C1* (SCO3817–SCO3815) and *bkdA2B2C2* (SCO3831–SCO3829; Sprusansky et al., 2005). The *bkdA2B2C2* cluster demonstrated consistent upregulation across all genes, reaching up to 20.6-fold at 72^nd^ hour.

In contrast, genes associated with branched-chain amino acid biosynthesis were generally downregulated. Except for a 2.32-fold upregulation of *ilvC2* (SCO7154) at 72^nd^ hour, reduced expression levels were observed for *ilvB* (SCO5512), SCO4962 and SCO5529 at both time points. Additionally, SCO2008–SCO2012 operon, which encodes a high-affinity ABC transporter system responsible for the uptake of these amino acids (Świa̧tek-Połatyńska et al., 2015), was uniformly upregulated, with expression increases of up to 5.7-fold at 72^nd^ hour (Figure 6B, Supplementary Table S3).

##### 3.5.3.3 N-acetylglucosamine/diacetylchitobiose transport systems

NgcE (SCO6005), NgcF (SCO6006), and NgcG (SCO6007) are components of an ABC transporter system that is involved in the N-acetylglucosamine (GlcNAc) uptake in *S. coelicolor* A3(2) (Świa̧tek-Połatyńska et al., 2015), showed increased expression in the Sco-pRA*lon* strain, reaching up to 3.7-fold at 72^nd^ hour.

Another transport system that exhibited significant expression changes is the DASABC sugar transport system, which is responsible for the uptake of diacetylchitobiose (GlcNAc)_2_ (Saito et al., 2007). Within this system, the structural gene *dasA* (SCO5232) was downregulated by approximately 2.5-fold at 24^th^ hour of fermentation. Similarly, the permease subunit gene *dasB* (SCO5233) showed a 3.37-fold downregulation at 24^th^ hour; however, this repression shifted to a comparable level of upregulation (3.25-fold) at 72^nd^ hour (Figure 6B, Supplementary Table S3).

##### 3.5.3.4 Phosphate metabolism

Consistent downregulation of genes involved in the regulation of phosphate metabolism was observed in the recombinant strain. This included the two-component PhoR (SCO4229)/PhoP (SCO4249) system and the regulatory proteins PhoU (SCO4228) and PhoX (SCO3790); among these genes, the strongest downregulation reached ~9.5-fold at 24^th^ hour and ~5.3-fold at 72^nd^ hour.

Significant transcriptional changes were also identified in genes associated with inorganic phosphate (Pi) uptake systems in *S. coelicolor*. All genes within the high-affinity Pi transport operon *pstSCAB* (SCO4139–SCO4142; Martín and Liras, 2021) exhibited a strong downregulation of approximately 20-fold exclusively at 24^th^ hour. Notably, the repression of SCO4139, which encodes PstB, was also validated by RT-qPCR analysis (Figure 7).

In contrast, the gene encoding the low-affinity Pi uptake system component *pitH2* (SCO1845; Santos-Beneit et al., 2008) showed a biphasic pattern, with ~2.12-fold downregulation at 24^th^ hour followed by marked upregulation (~9.42-fold) at 72^nd^ hour. Similarly, the putative orthologous *pst* operon (SCO6814–SCO6816; Lewis et al., 2010; Díaz et al., 2013) showed no significant change at 24^th^ hour but displayed consistent upregulation ranging from 6.73- to 9.4-fold at 72^nd^ hour. Finally, SCO4152, encoding a secreted 5′-nucleotidase (Martín et al., 2012), showed an approximately 2.7-fold increase in expression at 72^nd^ hour (Figure 6, Supplementary Table S3).

##### 3.5.3.5 Energy metabolism

RNA-seq analyses revealed significant alterations in the expression of genes related to energy metabolism in the recombinant strain compared to the wild type (Supplementary Table S3). Notably, the *nuo* operon (*nuoA–N*), which encodes the subunits of NADH dehydrogenase/Complex I (a key component of oxidative phosphorylation) in *S. coelicolor* (Brekasis and Paget, 2003) was strongly upregulated up to four-fold at both 24^th^ and 72^nd^ hours, indicating enhanced respiratory activity and elevated cellular energy generation in the recombinant strain.

DEGs related to fatty acid metabolism were also analyzed (Figure 6B, Supplementary Table S3). Genes involved in fatty acid degradation displayed a predominantly upregulated profile. Consistent increases at the 24^th^ and/or 72^nd^ hours were observed for SCO0364, SCO1838, SCO6026, SCO6071, SCO6732, and SCO6967. Of these genes, only SCO6967, which encodes a beta-ketoadipyl-CoA thiolase, showed negative regulation at the 72^nd^ hour. In contrast, DEGs associated with fatty acid biosynthesis did not exhibit a consistent expression pattern. At the 24^th^ hour, *fabB2* (SCO0548), *fasR* (SCO2386), and *fabF3* (SCO3248) were upregulated, indicating transient activation of lipid biosynthesis. However, at 72^nd^ hour, repression was observed for all detected genes (SCO0428, SCO1815, SCO1831, SCO2386, and SCO2387), except for *fabF3*, which remained upregulated.

Particularly notable was the downregulation of *fabD* (SCO2387), a core component of the primary fatty acid biosynthesis operon (*fabDHPF*; Zhang et al., 2020), together with repression of *fasR* (SCO2386), the transcriptional activator of this operon (Arabolaza et al., 2010). This coordinated repression suggests a metabolic shift away from fatty acid biosynthesis toward fatty acid catabolism, likely supporting increased energy production and precursor redistribution required for secondary metabolite biosynthesis.

#### 3.5.4 Morphological differentiation

A significant number of well-characterized genes associated with the *Streptomyces* life cycle were found to be differentially expressed in the Sco-pRA*lon* strain (Figure 6C, Supplementary Table S3). Genes involved in the transition from spore germination to vegetative mycelium were upregulated, including *filP* (~3.1-fold at 72^nd^ hour) and *scy* (~2.3-fold at 24^th^ hour). *bldM* and *bldN* genes, which are required for the initiation of the transition from vegetative growth to aerial mycelium formation (Merrick, 1976), were upregulated by approximately four-fold at 72^nd^ hour in the recombinant strain. Similarly, the *bldK* operon genes (SCO5112-5115), which encode an ABC transporter system essential for aerial hyphae development (Nodwell et al., 1996), were downregulated by up to three-fold at 24^th^ hour but showed increased expression of up to 4.5-fold at 72^nd^ hour of fermentation.

In *Streptomyces* species, chaplin (ChpA–H), rodlin and SapB proteins are known to play key roles in the formation of the hydrophobic sheath, a crucial structure in aerial mycelium differentiation (Claessen et al., 2003; Elliot et al., 2003). In the recombinant strain, these genes were notably upregulated. At 24^th^ hour of fermentation, expression of *chpG*, and *chpH* genes increased 2.34- and 2.72-fold, respectively, while *chpE* was downregulated by approximately 2.7-fold. At 72^nd^ hour, most *chp* genes exhibited up to 5.3-fold upregulation, except *chpA, chpF*, and *chpD*. The *ramCSAB* gene cluster (SCO6681-6685), responsible for SapB synthesis (O'Connor and Nodwell, 2005; Flärdh and Buttner, 2009), was upregulated up to 4.7-fold at 24^th^ hour and up to 13.6-fold at 72^nd^ hour. Consistently, the cluster regulator *ramR* (O'Connor and Nodwell, 2005), showed increased expression at both time points.

The *whi* (white) genes are involved in the differentiation of aerial mycelium into spore chains (Chater, 1972). Of these genes, *whiB, whiD, whiG*, and *whiI* showed no significant change at 24^th^ hour, but exhibited up to an 49.3-fold downregulation at 72^nd^ hour of fermentation.

Expression changes have also been observed in the DEGs associated with the septation and maturation of spore chains in the recombinant strain. Among members of the SsgA-like proteins (SALPs), which regulate sporulation-specific cell division (Traag and van Wezel, 2008), *ssgF* was downregulated by approximately 5.2-fold at 24^th^ hour whereas *ssgB* was upregulated at both time points (~4.5-fold at 24^th^ hour and ~2.7-fold at 72^nd^ hour). *ssgA* showed an approximately 2.1-fold increase only at 72^nd^ hour. During septation, genes responsible for proper chromosome segregation (Vollmer et al., 2019), *parB* (~2.5-fold up) and *sffA* (up to ~21.4-fold down), showed significant expression changes only at 72^nd^ hour of fermentation. For the gene *sarA*, which is associated with suppression of sporulation (Ou et al., 2008), approximately 2.5-fold increase in expression was detected at 72^nd^ hour. Additionally, *sapA*, encoding a spore-associated protein precursor (Yagüe et al., 2014), was upregulated by ~12.5-fold at 24^th^ hour, as confirmed by RT-qPCR (Figure 7). Finally, *hyaS*, involved in hyphal contact and pellet morphology (Koebsch et al., 2009), was upregulated by ~2.6-fold at 72^nd^ hour (Figure 6C, Supplementary Table S3).

### 3.6 Validation of transcriptome data using RT-qPCR

Using RT-qPCR, we measured the expression levels of 12 differentially expressed genes in the Sco-pRA*lon* strain (Supplementary Table S2). We then compared these expression levels with the corresponding RNA-seq measurements to validate our results. Figure 7 illustrates this comparison for several genes, including those critical for primary metabolism (SCO4659, *rpsL*; SCO4139, *ptsB*), secondary metabolism (SCO2518, *ecrA*2; SCO5893, *redK*; SCO5074, *act*VI-3), stress response (SCO0600, *sig*B; SCO0379, *cat*A), and morphological differentiation (SCO0409, *sap*A; SCO1675, *chp*H). We also included three randomly selected DEGs with high negative or positive abundance values in this comparison: SCO6577 (conserved hypothetical protein SC3F9.12), SCO6214 (putative permease), and SCO4878 (putative glycosyltransferase). At 24^th^ hour, six genes showed upregulation, while three exhibited downregulation (Figure 7A). Five of the seven maintained their elevated expression at 72^nd^ hour, along with SCO1675 and SCO6577, which were not detected at the earlier time point (Figure 7B). Conversely, only one of the downregulated genes displayed consistent downregulation (Figure 7B). We observed a high degree of concordance between the RT-qPCR and RNA seq results for all genes tested, with a Pearson correlation coefficient of >0.9 (Figures 7C, D), suggesting that the transcriptomic measurements are valid.

### 3.7 Effect of *lon* overexpression on *S. coelicolor* morphogenesis in liquid and solid cultures

**Figure 8:**
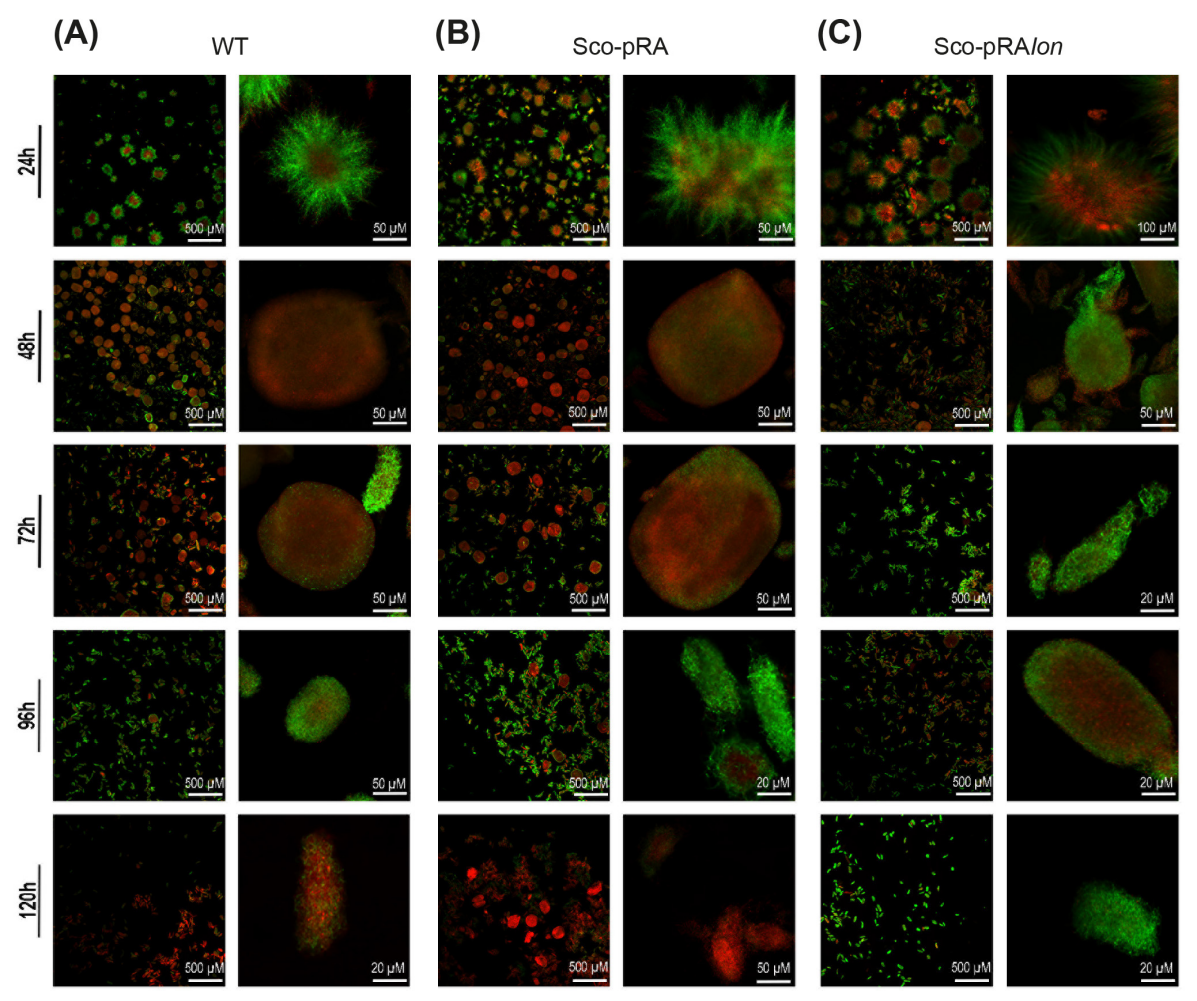
CLSM results of the wild type **(A)**, Sco-pRA **(B)**, and Sco-pRA*lon* **(C)** strains grown in R2YE medium. The cells were stained with SYTO 9 (green, live) and propidium iodide (red, dead). Each culture is shown as a merged wide-field image representing at different scales, as indicated by the scale bar in the figures.

Using CLSM, we examined the relationship between morphological transitions associated with the MI and MII stages and the live/dead cell dynamics of the recombinant strain and the two control strains (wild-type and Sco-pRA) under liquid culture conditions (Figure 8). Throughout the fermentation period, the control strains exhibited a developmental pattern highly consistent with the classical *Streptomyces* life cycle. However, the morphology of Sco-pRA*lon* differed markedly (Figure 8). In liquid culture, MI mycelia form pellets that grow radially and undergo PCD, dying from the center outward (Manteca et al., 2008). This process results in the formation of mycelial pellets containing dead (red) MI mycelia at the center and live (green) MI mycelia around them, as observed in all strains at the 24^th^ hour of fermentation (Figure 8). However, unlike the control strains, the recombinant strain exhibited a wider and more distinct dead cell area observed at the center of the pellets, indicating that it entered the PCD process at an earlier stage of development (Figure 8C). Similar diameters and a high percentage of dead mycelium were observed in the control strains at the 48^th^ and 72^nd^ hours (Figures 8A, B). These results suggests that control strains likely completed the first phase of exponential growth, which corresponds to MI mycelial development, within 24–48-h time interval. Subsequently they entered a transient growth arrest phase, which is marked by the cessation of pellet diameter increase. At 72^nd^ hour of fermentation, the general trend in these cultures showed that the second (exponential) growth phase had begun. This phase was marked by the start of growth of live MII mycelium from the remaining MI mycelium segments, which led to an increase in the live portions of the pellets. Consistent with this process, by 96th hour of fermentation, the live colony ratio had become dominant in both control cultures. As the proportion of dead cells gradually increased during incubation, by the end of the 120-h period, the cultures were predominantly consisted of dead mycelial segments (Figures 8A, B).

On the other hand, the onset of MI-to-MII differentiation, a key event in antibiotic biosynthesis, was observed at 48^th^ hour in Sco-pRA*lon*. This indicates that Sco-pRA*lon* may have exited the growth arrest phase earlier than the control strains, likely between 24 and 48 h (Figure 8). In the recombinant strain, culture revitalization associated with MII mycelial development was observed at 72^nd^ hour (compared to 96th hour in the control), and the sustained presence of a dense viable population up to 120 h was particularly remarkable (Figure 8C). Additionally, across the culture, this strain exhibited a smaller and more homogeneous pellet morphology. Despite its deviation from the classical developmental cycle, it is evident that the accelerated morphological transition in the Sco-pRA*lon* strain significantly contributes to its high level of antibiotic production.

**Figure 9:**
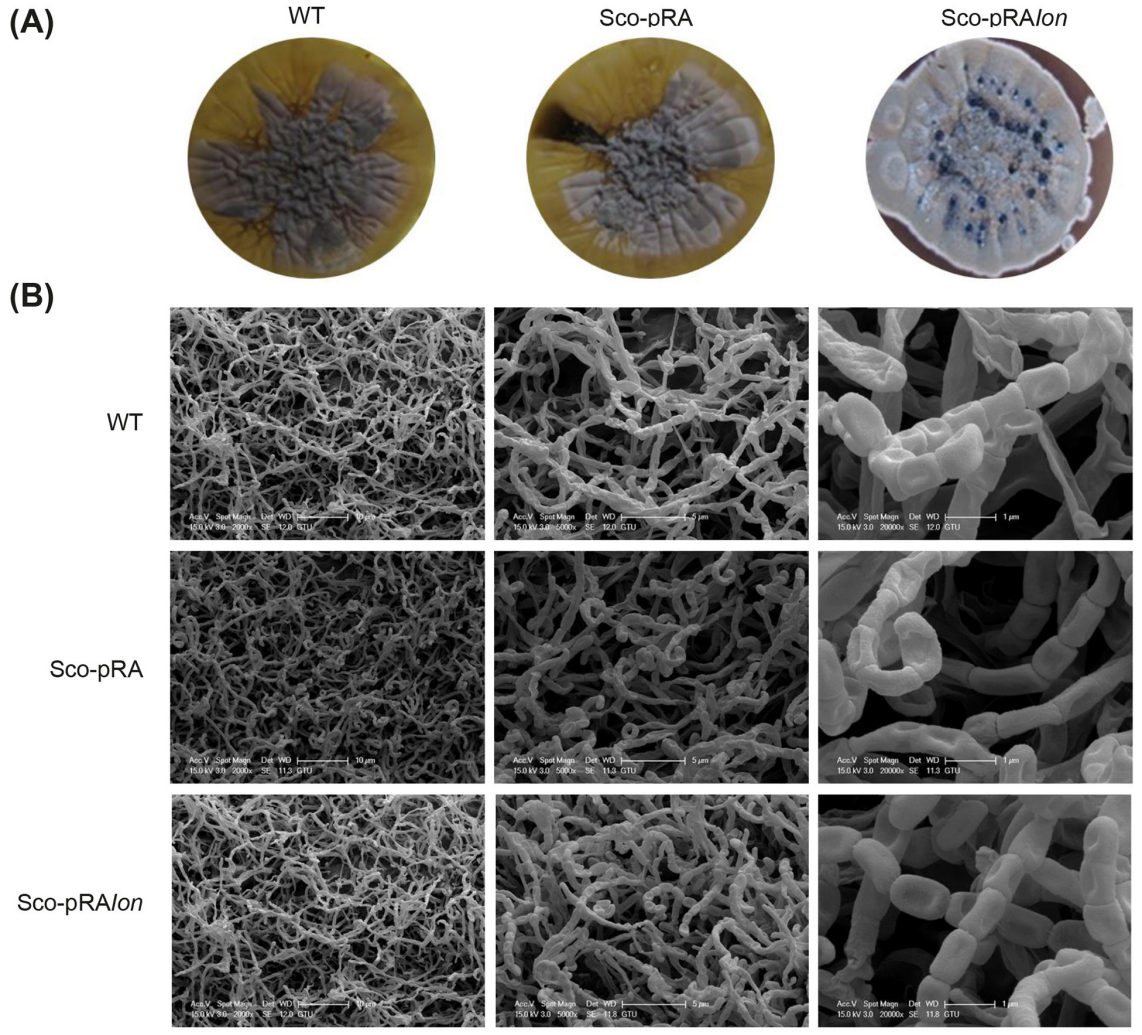
Macroscopic colony morphologies **(A)** and SEM micrographs **(B)** of *S. coelicolor* strains. For each strain, SEM micrographs are presented at three magnifications: 1 μm (left panel), 5 μm (middle panel), and 10 μm (right panel).

We then performed SEM analysis to investigate the morphological differentiation processes of *S. coelicolor* strains grown in solid culture (Figure 9A). After a 10-day incubation period, we observed that all strains underwent a PCD process, which involves the transformation of vegetative MI mycelia into aerial MII mycelia, which then form unigenomic spore chains. The wild-type and Sco-pRA strains, which displayed a similar developmental profile, were found to have spore chains with predominantly empty septa after 10 days incubation. In contrast, the Sco-pRA*lon* strain exhibited a significantly higher spore density. Additionally, the spore chains, which had a more coiled structure, demonstrated a more regular and healthier morphology (Figure 9). Macroscopic observations, carried out in parallel with the SEM analysis, revealed a noticeable increase in the production of gray-colored spores and blue actinorhodin droplets in the recombinant strain compared to the control strains (Figure 9). The results of all morphological analyses consistently showed that the presence of two additional copies of the *lon* gene in the recombinant strain resulted in the developmental changes that increased its capacity for secondary metabolite production.

## 4 Discussion

*Streptomyces* species are unique microorganisms distinguished by their capacity to synthesize a wide range of valuable bioactive compounds, which are of significant value for both medical and industrial applications (Donald et al., 2022). Lon protease, a key component of stress response regulons (Sobczyk et al., 2002) and a regulator of cellular differentiation (Tsilibaris et al., 2006; Kirthika et al., 2023), has emerged as a potential modulator of secondary metabolite production in bacteria. A previous study by our group showed that the *S. coelicolor* A3(2) recombinant strain that overexpresses the *lon* gene on an integrative vector (Sco-pRA*lon*) produces substantially higher levels of actinorhodin and undecylprodigiosin than the wild type (Demir et al., 2019). In this study, we used a combined approach of WGS, RNA-seq, SEM, and CLSM analyses to investigate the molecular and morphological mechanisms by which Lon protease overexpression triggers antibiotic biosynthesis in the recombinant strain Sco-pRA*lon*.

### 4.1 WGS revealed a novel *attB* site in the *S. coelicolor* genome

Integrative vectors are known to insert efficiently into the canonical *attB* region located within the *SCAC2.06c* (SCO3798) gene, which is shown to be non-essential for viability, approximately 300 times more effectively than into the three previously characterized pseudo-*attB* sites (*pseB1*, dihydropteroate synthase; *pseB2*, hypothetical protein; and *pseB3*, aspartate aminotransferase genes; Combes et al., 2002). WGS revealed that the pRA vector was found to be integrated into SCO3798 gene, as expected. However, the recombinant pRA*lon* vector integrated into not only the *attB* site within SCO3798, but also into an additional locus within the SCO3793 gene. According to StrepDB (https://strepdb.streptomyces.org.uk), the SCO3793 gene encodes a conserved hypothetical protein. This previously unreported insertion site represents a new *attB* locus and suggests the presence of alternative integration sites within the *S. coelicolor* genome. Database comparisons indicate that SCO3793 (StrepDB, 2025) is 60.5% similar to the uncharacterized YjqA protein of *Bacillus subtilis*. However, the *yjqA* gene was identified solely through genomic annotation and has no known function (Kunst et al., 1997). Therefore, insertions into SCO3798 and SCO3793 are theoretically not expected to directly affect antibiotic biosynthesis. However, it has also been reported that the location and copy number of chromosomal genes can affect expression levels in a non-linear manner (Schmid and Roth, 1987; Thompson and Gasson, 2001; Bryant et al., 2014; Loehlin and Carroll, 2016). Nevertheless, to more clearly disentangle Lon-specific effects from those related to integration number and gene dosage, further studies employing single-copy controlled integration systems are required. Additionally, as extra plasmid copies are known to cause metabolic stress in bacterial cells, dual integration of pRA*lon* may create indirect molecular consequences (Wang et al., 2006; Silva et al., 2012). While the pRA vector is not a high-copy-number plasmid capable of contributing to secondary metabolite activation, further research is clearly needed to shed light on this issue.

### 4.2 Global transcriptomic insights into Lon overexpression in *S. coelicolor*

The genes associated with four major biological processes including secondary metabolism, primary metabolism, stress response and morphological differentiation were differentially expressed in Sco-pRA*lon* (Figures 5, 6, Supplementary Table S3). Furthermore, GO and KEGG enrichment analyses indicated that numerous metabolic pathways are reprogrammed through Lon protease activity, with prominent enrichments observed in pathways directly linked to secondary metabolite biosynthesis (Figures 3–5). These findings support our previous studies (Demir et al., 2019; Barkad et al., 2021; Genel and Tunca, 2024; Yilmaz et al., 2025) by demonstrating that Lon protease plays a multifaceted regulatory role at the cellular level.

#### 4.2.1 Secondary metabolism

In fully sequenced genomes of the genus *Streptomyces*, genes responsible for secondary metabolite production are organized into biosynthetic clusters ranging from a few kilobases to over 100 kb (Bentley et al., 2002). At least five antibiotics with distinct chemical characteristics are synthesized in *S. coelicolor*, including actinorhodin (ACT), undecylprodigiosin (RED), the polyketide CPK, the calcium-dependent antibiotic (CDA), and methylenomycin (Hopwood, 1999). In the present study, *lon* overexpression was associated with pronounced transcriptional activation across several of these clusters, particularly ACT, RED, and CDA. Moreover, activation extended beyond major antibiotic pathways to include additional secondary metabolite clusters such as coelibactin, coelichelin, melanin, geosmin, SapB, hopanoids, and 2-methylisoborneol, indicating a broad regulatory impact.

Differential expression was not limited to biosynthetic clusters but also involved pathway-specific regulators, two-component systems (TCSs), and global transcription factors. The marked upregulation of key activator genes (*actII-ORF4, redD, cdaR, rapA2, ecrA1/A2* etc.), supports the notion that Lon overexpression enhances antibiotic production through stimulation of positive regulators (Lu et al., 2007; Yong-quan et al., 2004). Conversely, the expression of global repressor gene *das*R (Rigali et al., 2008) and the *abrB1* gene, which encodes a component of the AbrB1/B2 TCS that negatively regulates ACT and RED biosynthesis (Sánchez de la Nieta et al., 2020), were found to be downregulated. The downregulation of these repressor genes indicates relief of transcriptional repression of antibiotic biosynthetic clusters. These findings align well with the phenotypic results, which demonstrated markedly increased antibiotic production in the Sco*-*pRA*lon* strain (Demir et al., 2019).

#### 4.2.2 Stress response

Transcriptomic analysis of the Sco*-*pRA*lon* strain revealed pronounced activation and transcriptional reprogramming of cellular stress responses. Specifically, genes encoding universal stress proteins (USPs) and several sigma factors homologous to the general stress response factor σ(B) of *B. subtilis* were upregulated. In parallel, induction of SOS response genes (*uvrA, lexA, recA*, and SCO0695) indicates activation of DNA damage–associated repair mechanisms and supports the idea that Lon contributes to enhanced tolerance against genotoxic stress. This observation is consistent with previous reports demonstrating that Lon is required for an effective SOS response in bacteria (Breidenstein et al., 2012; Kirthika et al., 2023). The *S. coelicolor* recombinant strain (Sco-Lon), which expresses the *lon* gene on a high-copy pSPG vector, exhibited a higher survival rate following UV exposure than control strains (Yilmaz et al., 2025), thereby supporting our results.

In addition to genotoxic stress, the Sco-pRA*lon* strain appears to display enhanced tolerance to osmotic stress. Upregulation of sigma factors associated with the osmotic stress response, including σM, which acts downstream of σB in the regulatory cascade (Lee et al., 2005), suggests transcriptional reprogramming toward osmoprotective pathways. Comparable phenotypes have been reported in other bacteria, where *lon* overexpression improved osmotic stress tolerance in *Escherichia coli, D. solani*, and *B. thuringiensis* (Figaj et al., 2020; Barkad et al., 2021). Consistent with these findings, osmotic stress has been shown to induce *lon* expression in *D. dadantii* (Jiang et al., 2016). In agreement with these findings, our previous study demonstrated that Sco-Lon strain, which overexpress *lon* gene on a high copy number plasmid, displays improved growth under high KCl concentrations compared to controls (Yilmaz et al., 2025).

Upregulation of oxidative stress related genes suggests that the recombinant strain experiences oxidative stress and that Lon may indirectly activate oxidative stress–related metabolic pathways. Consistently, studies in *Salmonella typhimurium* have shown that *lon* mutants exhibit a reduced capacity to neutralize free radicals under oxidative stress (Kirthika et al., 2020). These findings underscore Lon's central role in managing responses to abiotic stressors, including cold, acidic, and osmotic conditions.

Collectively, these findings suggest that Lon is a component of a cellular defense network activated in response to a variety of environmental stressors in *S. coelicolor*. However, previous studies have shown that excessive *lon* expression in high-copy-number or strongly induced systems may impair cell growth due to increased proteolytic activity (Goff and Goldberg, 1987; Christensen et al., 2004; Tsilibaris et al., 2006). Conversely, in recombinant *B. thuringiensis* and *S. coelicolor* strains that acquire resistance to different stress conditions, the *lon* gene has been expressed from multicopy vectors without causing significant cellular damage (Barkad et al., 2021; Yilmaz et al., 2025). These seemingly divergent observations suggest that the physiological impact of Lon overexpression may depend on host background, expression levels, and the nature of the stress context. Therefore, the potential consequences of *lon* overexpression on cellular homeostasis and growth should be carefully assessed in a strain- and system-specific manner.

#### 4.2.3 Primary metabolism

##### 4.2.3.1 Ribosomal protein synthesis

In *Streptomyces* species, the transient growth arrest observed under submerged culture conditions, which occurs prior to antibiotic production, corresponds to the transition from vegetative to multinucleated, differentiated mycelium. This developmental shift is frequently associated with repression of the ribosomal protein synthesis (Blanco et al., 1994; Manteca et al., 2008).

The DEG analysis revealed that the majority of genes encoding both large (Rpl) and small (Rps) ribosomal subunits were significantly downregulated in the Sco-pRA*lon* strain at 24^th^ hour of fermentation. These transcriptomic findings are consistent with confocal microscopy observations, which revealed accelerated morphological differentiation in the recombinant strain compared to controls (Figure 8). This suggests that translational processes are suppressed at an earlier developmental stage in the recombinant strain.

Lon protease has been reported to degrade ribosomal proteins during stringent response, thereby contributing to the generation of free amino acids that can be redirected toward synthesis of stress-adaptation enzymes (Kuroda, 2006). Accordingly, the reduced expression of ribosomal genes observed may indirectly reflect Lon-dependent proteolytic activity, linking Lon overexpression to resource reallocation during early differentiation and activation of secondary metabolism.

##### 4.2.3.2 Branched-chain amino acid metabolism

In *S. coelicolor*, up to 50% of the acetate required for the biosynthesis of the type II polyketide actinorhodin is derived from the catabolism of branched-chain amino acids (Stirrett et al., 2009). In Sco-pRA*lon*, genes within the *bkdA2B2C2* cluster, which is responsible for BCAA degradation, were consistently upregulated. This observation aligns with the ensemble modeling analyses by Genel and Tunca (2024), which predicted that *lon* overexpression enhances the metabolic flux through the *bkdA2B2C2* pathway.

In parallel, the operon encoding the ABC transporter responsible for BCAA uptake showed clear transcriptional activation, suggesting an increased intracellular BCAA concentration. This increase may be partially attributed to increased PCD and cellular lysis in the recombinant strain, processes that releases proteins and free amino acids into the extracellular environment and promote nutrient recycling. Taken together, in the Sco-pRA*lon* strain, characterized by pronounced transcriptional and phenotypic stress signatures, increased intracellular BCAA pools likely serve as critical metabolic precursors that support and sustain the elevated actinorhodin production phenotype.

##### 4.2.3.3 N-acetylglucosamine signaling

The expression of *dasR*, a key negative regulator of antibiotic biosynthesis, was markedly downregulated in the recombinant strain at 72^nd^ hour of fermentation. According to the established DasR regulon model in *S. coelicolor*, DasR represses *actII-ORF4* and *redZ* by binding to their promoters under nutrient-rich conditions, thereby inhibiting ACT and RED biosynthesis (Van Wezel and McDowall, 2011; Świa̧tek-Połatyńska et al., 2015). During nutrient limitation, however, PCD releases N-acetylglucosamine (GlcNAc), which is converted intracellularly to glucosamine-6-phosphate (GlcN-6P). This metabolite binds to DasR, preventing its interaction with DNA and relieving repression of secondary metabolic and developmental genes. Supporting this mechanism, the addition of GlcNAc to minimal medium has been shown to induce both sporulation and ACT/RED biosynthesis in *S. coelicolor* (Świa̧tek-Połatyńska et al., 2015).

Consistent with these findings, the Sco-pRA*lon* strain exhibited transcriptional activation of *ngcE, ngcF, ngcG*, and *dasB*, genes encoding transporters involved in the uptake of GlcNAc and its chitin-derived dimer, diacetylchitobiose (GlcNAc)_2_. The observed expression changes across the DasR regulon may thus contribute to the prolonged antibiotic production and altered developmental trajectory observed in this strain up to 120 h of fermentation.

##### 4.2.3.4 Phosphate metabolism

*Streptomyces* species possess complex regulatory networks that coordinate nutrient adaptation. One such network is the PhoR-PhoP two-component system, which governs the PHO regulon under phosphate limiting conditions (Sola-Landa et al., 2003). In *S. coelicolor*, phosphate uptake is primarily mediated by the high-affinity ABC transporter, *pstSCAB*, whose expression responds strongly to extracellular phosphate levels (Martín and Liras, 2021).

In the Sco-pRA*lon* strain, *phoR, phoP*, and *phoU* expression was markedly downregulated (up to 9.5-fold), accompanied by strong repression of the *pstSCAB* operon (up to 20-fold) and reduced expression of the extracellular phosphatase *phoX*. These data suggest suppression of the PHO regulon and are consistent with phosphate-replete conditions (Hsieh and Wanner, 2010; Virolle, 2020). Nevertheless, despite the lack of external phosphate limitation, the Sco-pRA*lon* strain displayed high ACT and RED production, similar to the phenotype of Δ*phoP* and Δ*phoR*–*phoP* mutants (Sola-Landa et al., 2003). This apparent paradox may reflect reduced intracellular phosphate availability rather than extracellular scarcity and is potentially linked to elevated ATP consumption driven by Lon's ATPase activity. This energy imbalance likely triggered adaptive metabolic rewiring, which is consistent with reports that secondary metabolite synthesis in *Streptomyces* is favored under conditions of low intracellular ATP (Martín, 2004; Esnault et al., 2017).

PCD during development may further contribute to phosphate dynamics. In *Streptomyces*, the autolysis of MI mycelia releases amino acids, nucleotides, and phosphates to support the surviving population (Barka et al., 2016; Virolle, 2020). In Sco-pRA*lon*, the strong PCD observed at 24^th^ hour aligns with the increased expression of the CDA and RED clusters, which are both known to induce membrane damage and lysis. This suggests an enhanced release of phosphate into the medium. Concurrent induction of the extracellular 5'-nucleotidase gene SCO4152 supports the hypothesis of nucleotide hydrolysis and liberation of inorganic phosphate; however, metabolic validation is required.

Significant upregulation was seen in the expression of *pitH2* (encoding low-affinity phosphate transporter) and the genes belonging to the putative *pst* operon ortholog (SCO6814–SCO6816) in *S. coelicolor* at the 72^nd^ hour. Meanwhile, the expression of *pstSCAB* genes was suppressed at 24^th^ hour and did not undergo significant change at 72^nd^ hour. These results suggest a shift in time from energy-intensive, ATP-driven Pst transport to lower-energy proton symporter systems, such as Pit, with alternative phosphate uptake routes mediated by the orthologous *pst* operon being engaged over time. This shift reflects an adaptation strategy that conserves ATP. Given the ATPase activity of Lon protease and *lon*'s dual genomic integration in Sco-pRA*lon*, this metabolic rerouting likely mitigates intracellular energy stress while maintaining phosphate homeostasis.

Together, these phosphate-dependent adaptations not only reflect a restructured nutrient-sensing network but also suggest broader shifts in cellular energy balance, prompting a deeper examination of how Lon overexpression influences central energy metabolism pathways in *S. coelicolor*.

##### 4.2.3.5 Energy metabolism

Transcriptomic analyses revealed that the Sco-pRA*lon* strain compensates for ATP deficiency by activating oxidative energy metabolism. Genes encoding the nuo operon, which constitutes NADH dehydrogenase (Complex I) of the electron transport chain (ETC), were strongly upregulated, indicating enhanced oxidative phosphorylation. These data suggest that the recombinant strain undergoes a compensatory metabolic shift favoring oxidative phosphorylation as the primary ATP recovery strategy.

Furthermore, lipid metabolism appeared to play a complementary role in maintaining energy homeostasis. The Sco-pRA*lon* strain displayed coordinated upregulation of genes associated with triacylglycerol (TAG) degradation, as well as the downregulation of genes involved in fatty acid biosynthesis within the *fabDHPF* operon. This pattern reflects the redirection of acetyl-CoA from lipid storage to the TCA cycle to sustain oxidative metabolism. Similar TAG catabolism has been reported in *S. lividans ppk* mutants under ATP limitation, where lipid turnover replenishes intracellular energy pools (Esnault et al., 2017).

The activation of oxidative metabolism, however, leads to increased production of reactive oxygen species (ROS) and oxidative stress. The upregulation of catalase genes (*katA, cpeB* and *katA2*) and the increased biosynthesis of actinorhodin (ACT), an antioxidant secondary metabolite, indicate that redox homeostasis is tightly coupled to metabolic adaptation. ACT has been shown to scavenge excess electrons from the respiratory chain and neutralize ROS, simultaneously regulating oxidative stress and respiratory efficiency (Esnault et al., 2017; Virolle, 2020).

The relationship between ATP stress, oxidative metabolism, and antibiotic biosynthesis is consistent with previous findings in *Streptomyces* mutants with altered ATP homeostasis. In *S. lividans*, ATPase overexpression reduced intracellular ATP levels, decreased lipid content, and induced ACT, RED, and CDA biosynthesis (Seghezzi et al., 2022). Similarly, artificial ATP spilling via the DX system, which dephosphorylates ATP into ADP, in *S. albogriseolus/viridodiastaticus* activated oxidative metabolism, increased ATP production, and stimulated antioxidant metabolite synthesis (Apel et al., 2023).

Together, these results indicate that *lon* overexpression drives a systemic reprogramming of energy metabolism. This is accomplished by enhancing oxidative phosphorylation, mobilizing lipid reserves, and coupling oxidative stress responses with secondary metabolite production. Thus, a tight regulatory link is established between energy balance, redox adaptation, and antibiotic biosynthesis in *S. coelicolor*.

#### 4.2.4 Morphological differentiation

*S. coelicolor* is widely used as a model organism for studies on the regulation of antibiotic production and morphological differentiation. It exhibits a complex life cycle that includes PCD and sporulation (Yagüe et al., 2013; Barka et al., 2016). Although the developmental dynamics of solid and liquid cultures differ, transcriptomic and proteomic studies have revealed greater similarities in morphogenesis (Manteca et al., 2010a; Yagüe et al., 2014). A prominent shared feature is the coordinated upregulation of genes and proteins associated with secondary metabolism in MII mycelia (Manteca et al., 2010a,b; Yagüe et al., 2013, 2014).

In this study, transcriptomic analysis of the Sco-pRA*lon* strain revealed extensive developmental and regulatory reprogramming relative to the wild type. At 72^nd^ hour, a wide range of genes involved in aerial mycelium formation, including multiple *bld* genes and most *chp* (chaplin) genes were upregulated. Given that chaplins are essential structural determinants of pellet architecture, as demonstrated in the *S. coelicolor* M145 Δ*chpABCDEFGH* mutant (Van Veluw et al., 2012), their upregulation strongly indicates that *lon* overexpression directly remodels pellet morphology. In parallel, the *ram* cluster, responsible for the biosynthesis of SapB (a lantibiotic-like morphogen reducing surface tension; Kodani et al., 2004), was also upregulated. This finding provides further molecular evidence for altered mycelial organization and supports a mechanistic link between Lon overexpression, morphological changes, and activation of secondary metabolism.

Conversely, the genes associated with sporulation, including *whiB, whiG, whiI, whiD, ssgF*, and *ssfA*, and late sporulation sigma factor, *sigF*, were downregulated. This transcriptional pattern indicates developmental arrest prior to sporulation and suggests that cellular resources are preferentially redirected toward mycelial differentiation rather than spore maturation.

On the other hand, several genes associated with hydrophobic sheath formation and sporulation (*parB, ssgA, ssgB, sarA*, and *sapA*) were found to be upregulated in Sco-pRA*lon*. Consistent with our findings, Yagüe et al. demonstrated that during the MII mycelium stage of *S. coelicolor*, genes regulating hydrophobic cover formation and sporulation are also activated even in non-sporulating liquid cultures (Yagüe et al., 2014). Accordingly, our SEM and macroscopic observations confirmed increased spore density, more organized spore-chain architecture, and increased production of gray spores and blue actinorhodin droplets in the recombinant strain. Lon overexpression also impacted apical growth. The upregulation of FilP and Scy, which are key components of the TIPOC complex that controls hyphal tip organization in submerged cultures (Holmes et al., 2013; Van Dissel et al., 2014), indicates sustained apical growth and modified filament growth dynamics. Additional upregulation of pellet formation and stability genes (*ssgA, hyaS*, and *scy*) further supports structural remodeling. HyaS maintains hyphal contacts during submerged growth (Koebsch et al., 2009), and coordinated Scy-ParA activity supports organized pellet architecture (Ditkowski et al., 2013). Notably, elevated *ssgA* expression aligns with prior reports linking its overexpression to improved growth rates, reduced lag phase, smaller mycelia, and improved fermentation productivity (Van Wezel et al., 2006). Collectively, these transcriptional changes suggest that Lon overexpression modulates morphogenetic networks controlling pellet formation and differentiation.

CLSM analysis provided physiological confirmation of these molecular findings. Sco-pRA*lon* undergoes PCD and MII development earlier than WT or Sco-pRA strains. Importantly, the viable cell population observed at the 72^nd^ hour persisted until the 120^th^ hour, along with small, homogeneous pellets. This extended viability indicates prolonged metabolic activity and coincides with the reported peak of actinorhodin biosynthesis (Demir et al., 2019).

*S. coelicolor* exhibits a heterogeneous pellet population consisting of both large and small pellets under liquid culture conditions (Van Veluw et al., 2012). Morphological heterogeneity observed in the control strains has been replaced by a more homogeneous, small-sized pellet population with high viability in the recombinant strain, where large pellets are rarely encountered. According to Van Wezel et al. (2006), cells at the center of large pellets experience greater oxygen and nutrient limitations than cells in smaller pellets. Thus, the small pellet morphology observed in the recombinant strain may confer an adaptive advantage by alleviating cellular stress. Consequently, Lon-mediated morphological optimization appears to alleviate mass-transfer constraints and promote conditions favorable for secondary metabolite production.

The macroscopic and physiological changes driven by *lon* overexpression are therefore directly associated with the activation of secondary metabolism. This finding is consistent with morphology-targeted approaches previously shown to enhance metabolite yields in *Streptomyce*s (Van Wezel et al., 2006; Dobson et al., 2008; Sohoni et al., 2012; López-García et al., 2014; Holtmann et al., 2017; Lajtai-Szabó et al., 2022). For example, immobilization of *S. coelicolor* in alginate beads increased the proportion of viable MII hyphae and boosted actinorhodin production (López-García et al., 2014), while reduced pellet diameter has been correlated with higher antibiotic yields (Dobson et al., 2008). Similarly, adding glass beads to the culture medium increased actinorhodin production by reducing pellet size in *S. coelicolor* (Sohoni et al., 2012; Holtmann et al., 2017).

In summary, our data collectively suggest that *lon* overexpression reprograms core primary metabolism in *S. coelicolor*, shifting the cell from a growth-focused metabolism to an energetically optimized metabolism. Early translational downregulation indicates a reduced biosynthetic load. Meanwhile, enhanced branched-chain amino acid catabolism and GlcNAc-mediated regulation likely supply key intermediates to central carbon metabolism. The repression of the PhoR–PhoP regulon and the transition from ATP-driven phosphate uptake to lower-energy Pit-based transport system suggest adjustments in phosphate and ATP homeostasis. Concurrent activation of the oxidative phosphorylation, and lipid turnover pathways indicates that oxidative metabolism compensates for the elevated ATP consumption associated with Lon activity. Taken together, Lon overexpression promotes a coordinated metabolic state that prioritizes energetic efficiency and resource redistribution within primary metabolism.

Beyond metabolism, *lon* overexpression accelerates developmental transitions, remodels pellet morphology, and prolongs metabolically active MII states. The early onset of PCD, rapid formation of MII, and extended MII viability collectively create a physiological landscape optimized for prolonged secondary metabolite biosynthesis. From an applied perspective, these findings highlight the potential of Lon-mediated genetic reprogramming as a promising strategy for rational morphological engineering and metabolic optimization under submerged industrial fermentation conditions.

**Figure 10:**
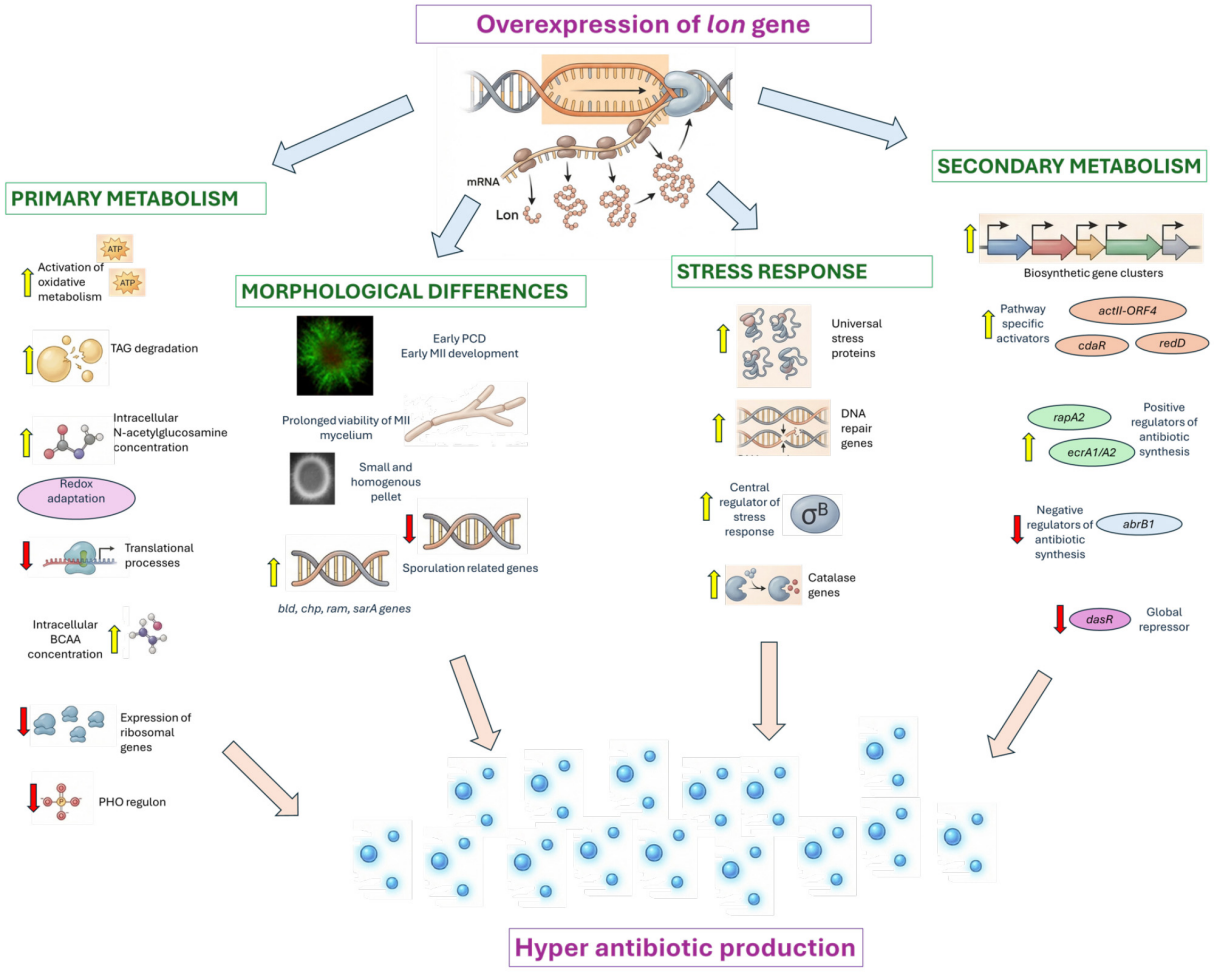
Schematic overview of Lon protease–mediated regulation of key physiological processes in *S. coelicolor*. The yellow and red arrows indicate genes that predominantly exhibit up- and downregulation trends, respectively, in the Sco-pRA*lon* strain.

As summarized in Figure 10, Lon protease exerts multifaceted and hierarchical control over metabolic and developmental networks. These insights may guide future efforts to engineer *Streptomyces* strains with enhanced metabolite production capacity.

## 5 Conclusion and future perspectives

This study demonstrates that overexpressing the *lon* gene reprograms the cellular physiology of *S. coelicolor*. This reprogramming affects not only the biosynthesis of secondary metabolites, but also critical biological processes, including morphological differentiation, stress response regulation, and primary metabolism. These findings position Lon as a central regulatory hub linking metabolic state, morphogenesis, and antibiotic production. Beyond clarifying Lon's physiological role, this work provide insight into the regulatory nodes that could be exploited to achieve elevated and robust antibiotic yields. The recombinant strain shows biotechnological promise, particularly considering the industrial potential of actinorhodin and other colored metabolites. Such natural pigments can serve as environmentally friendly alternatives to toxic synthetic dyes.

Nevertheless, further studies are required to resolve the mechanistic basis of Lon-dependent transcriptional reprogramming, such as identification of potential Lon protease substrates through quantitative proteomics and targeted validation approaches; systematic modulation of *lon* gene dosage using promoters of varying strengths or inducible expression systems; functional characterization of key regulatory nodes governing developmental and metabolic transitions. Moreover, given the evolutionary conservation of Lon protease across *Streptomyces* species, it remains to be determined whether Lon functions as a general activator of secondary metabolism in other industrially relevant strains.

Overall, this work establishes a valuable framework for Lon protease-guided strain engineering and provides a foundation for the rational design of high-value *Streptomyces* cell factories for medical and industrial applications.

## Data Availability

The datasets presented in this study can be found in the NCBI Sequence Read Archive (SRA; https://www.ncbi.nlm.nih.gov/sra), BioProject accession number PRJNA1077142.

## References

[B1] Alam K. Mazumder A. Sikdar S. Zhao Y. M. Hao J. Song C. . (2022). *Streptomyces*: the biofactory of secondary metabolites. Front. Microbiol. 13:968053. doi: 10.3389/fmicb.2022.968053 36246257 PMC9558229

[B2] Andrews S. (2010). FastQC: a quality control tool for high throughput sequence data. Babraham Bioinformatics, Cambridge, United Kingdom. Available online at: https://www.bioinformatics.babraham.ac.uk/projects/fastqc/ (Accessed March 10, 2026).

[B3] Apel C. Levasseur M. Lejeune C. Korch S. B. Guérard F. David M. . (2023). Metabolic adjustments in response to ATP spilling by the small DX protein in a *Streptomyces* strain. Front. Cell Dev. Biol. 11:1129009. doi: 10.3389/fcell.2023.1129009 PMC10030506 36968208

[B4] Arabolaza A. D'Angelo M. Comba S. Gramajo H. (2010). FasR, a novel class of transcriptional regulator, governs the activation of fatty acid biosynthesis genes in *Streptomyces coelicolor*. Mol. Microbiol. 78, 47–63. doi: 10.1111/j.1365-2958.2010.07274.x 20624224

[B5] Barka E. A. Vatsa P. Sanchez L. Gaveau-Vaillant N. Jacquard C. Klenk H.-P. . (2016). Taxonomy, physiology, and natural products of actinobacteria. Microbiol. Mol. Biol. Rev. 80, 1–43. doi: 10.1128/MMBR.00019-15 26609051 PMC4711186

[B6] Barkad M. A. Bayraktar A. Doruk T. Tunca S. (2021). Effect of lon protease overexpression on endotoxin production and stress resistance in *Bacillus thuringiensis*. Curr. Microbiol. 78, 3483–3493. doi: 10.1007/s00284-021-02610-w 34272975

[B7] Benjamini Y. Yekutieli D. (2001). The control of the false discovery rate in multiple testing under dependency. Ann. Stat. 1165–1188. doi: 10.1214/aos/1013699998

[B8] Bentley S. D. Chater K. F. Cerdeño-Tárraga A.-M. Challis G. L. Thomson N. R. James K. D. . (2002). Complete genome sequence of the model actinomycete *Streptomyces coelicolor* A3(2). Nature 417, 141–147. doi: 10.1038/417141a 12000953

[B9] Bibb M. J. (2005). Regulation of secondary metabolism in streptomycetes. Curr. Opin. Microbiol. 8, 208–215. doi: 10.1016/j.mib.2005.02.016 15802254

[B10] Blanco G. Rodicio M. R. Puglia A. M. Méndez C. Thompson C. J. Salas J. A. (1994). Synthesis of ribosomal proteins during growth of *Streptomyces coelicolor*. Mol. Microbiol. 12:375–385. doi: 10.1111/j.1365-2958.1994.tb01027.x 7545948

[B11] Bolger A. M. Lohse M. Usadel B. (2014). Trimmomatic: a flexible trimmer for illumina sequence data. Bioinformatics 30, 2114–2120. doi: 10.1093/bioinformatics/btu170 24695404 PMC4103590

[B12] Breidenstein E. B. M. Bains M. Hancock R. E. W. (2012). Involvement of the lon protease in the SOS response triggered by ciprofloxacin in *Pseudomonas aeruginosa* PAO1. Antimicrob. Agents Chemother. 56, 2879–2887. doi: 10.1128/AAC.06014-11 22450976 PMC3370746

[B13] Brekasis D. Paget M. S. B. (2003). A novel sensor of NADH/NAD+ redox poise in *Streptomyces coelicolor* A3(2). *EMBO J*. 22, 4856–4865. doi: 10.1093/emboj/cdg453 PMC212721 12970197

[B14] Bryant J. A. Sellars L. E. Busby S. J. W. Lee D. J. (2014). Chromosome position effects on gene expression in *Escherichia coli* K-12. Nucleic Acids Res. 42, 11383–11392. doi: 10.1093/nar/gku828 25209233 PMC4191405

[B15] Challis G. L. Hopwood D. A. (2003). Synergy and contingency as driving forces for the evolution of multiple secondary metabolite production by *Streptomyces* species. Proc. Natl. Acad. Sci. U.S.A. 100, 14555–14561. doi: 10.1073/pnas.1934677100 12970466 PMC304118

[B16] Chater K. F. (1972). A morphological and genetic mapping study of white colony mutants of *Streptomyces coelicolor*. Microbiology 72, 9–28. doi: 10.1099/00221287-72-1-9 4561048

[B17] Chen Y. Chen Y. Shi C. Huang Z. Zhang Y. Li S. . (2018). SOAPnuke: a MapReduce acceleration-supported software for integrated quality control and preprocessing of high-throughput sequencing data. Gigascience 7, 1–6. doi: 10.1093/gigascience/gix120 29220494 PMC5788068

[B18] Christensen S. K. Maenhaut-Michel G. Mine N. Gottesman S. Gerdes K. Van Melderen L. (2004). Overproduction of the Lon protease triggers inhibition of translation in *Escherichia coli*: involvement of the yefM–yoeB toxin–antitoxin system. Mol. Microbiol. 51, 1705–1717. doi: 10.1046/j.1365-2958.2003.03941.x 15009896

[B19] Claessen D. Rink R. de Jong W. Siebring J. de Vreugd P. Boersma F. G. H. . (2003). A novel class of secreted hydrophobic proteins is involved in aerial hyphae formation in *Streptomyces coelicolor* by forming amyloid-like fibrils. Genes Dev. 17, 1714–1726. doi: 10.1101/gad.264303 12832396 PMC196180

[B20] Combes P. Till R. Bee S. Smith M. C. M. (2002). The *Streptomyces* genome contains multiple pseudo-attB sites for the ϕC31-encoded site-specific recombination system. J. Bacteriol. 184, 5746–5752. doi: 10.1128/JB.184.20.5746-5752.2002 12270833 PMC139614

[B21] Demir Z. Bayraktar A. Tunca S. (2019). One extra copy of *lon* gene causes a dramatic increase in actinorhodin production by *Streptomyces coelicolor* A3(2). *Curr. Microbiol*. 76, 1045–1054. doi: 10.1007/s00284-019-01719-3 31214822

[B22] Díaz M. Sevillano L. Rico S. Lombo F. Braña A. F. Salas J. A. . (2013). High level of antibiotic production in a double polyphosphate kinase and phosphate-binding protein mutant of *Streptomyces lividans*. FEMS Microbiol. Lett. 342, 123–129. doi: 10.1111/1574-6968.12098 23398561

[B23] Ditkowski B. Holmes N. Rydzak J. Donczew M. Bezulska M. Ginda K. . (2013). Dynamic interplay of ParA with the polarity protein, Scy, coordinates the growth with chromosome segregation in *Streptomyces coelicolor*. Open Biol. 3, 1–13. doi: 10.1098/rsob.130006 23536551 PMC3718342

[B24] Dobson L. F. O'Cleirigh C. C. O'Shea D. G. (2008). The influence of morphology on geldanamycin production in submerged fermentations of *Streptomyces* hygroscopicus var. geldanus. Appl. Microbiol. Biotechnol. 79, 859–866. doi: 10.1007/s00253-008-1493-3 18443778

[B25] Donald L. Pipite A. Subramani R. Owen J. Keyzers R. A. Taufa T. (2022). *Streptomyces*: still the biggest producer of new natural secondary metabolites, a current perspective. Microbiol. Res. 13, 418–465. doi: 10.3390/microbiolres13030031

[B26] Elliot M. A. Karoonuthaisiri N. Huang J. Bibb M. J. Cohen S. N. Kao C. M. . (2003). The chaplins: a family of hydrophobic cell-surface proteins involved in aerial mycelium formation in *Streptomyces coelicolor*. Genes Dev. 17, 1727–1740. doi: 10.1101/gad.264403 12832397 PMC196181

[B27] Esnault C. Dulermo T. Smirnov A. Askora A. David M. Deniset-Besseau A. . (2017). Strong antibiotic production is correlated with highly active oxidative metabolism in *Streptomyces coelicolor* M145. Sci. Rep. 7:200. doi: 10.1038/s41598-017-00259-9 28298624 PMC5427975

[B28] Fernandez M. Sanchez J. (2001). Viability staining and terminal deoxyribonucleotide transferase-mediated dUTP nick end labelling of the mycelium in submerged cultures of *Streptomyces antibioticus* ETH7451. J. Microbiol. Methods 47, 293–298. doi: 10.1016/S0167-7012(01)00332-3 11714519

[B29] Figaj D. Czaplewska P. Przepióra T. Ambroziak P. Potrykus M. Skorko-Glonek J. (2020). Lon protease is important for growth under stressful conditions and pathogenicity of the phytopathogen, bacterium *Dickeya solani*. Int. J. Mol. Sci. 21:3687. doi: 10.3390/ijms21103687 32456249 PMC7279449

[B30] Flärdh K. Buttner M. J. (2009). *Streptomyces* morphogenetics: dissecting differentiation in a filamentous bacterium. Nat. Rev. Microbiol. 7, 36–49. doi: 10.1038/nrmicro1968 19079351

[B31] Genel N. Tunca S. (2024). Combined effect of polyphosphate kinase and lon protease in *Streptomyces coelicolor* A3(2) antibiotic production. Arch. Microbiol. 206:420. doi: 10.1007/s00203-024-04138-6 39331181

[B32] Goff S. A. Goldberg A. L. (1987). An increased content of protease La, the lon gene product, increases protein degradation and blocks growth in *Escherichia coli*. J. Biol. Chem. 262, 4508–4515. doi: 10.1016/S0021-9258(18)61221-9 3549709

[B33] Gur E. Biran D. Ron E. Z. (2011). Regulated proteolysis in Gram-negative bacteria-how and when? Nat. Rev. Microbiol. 9, 839–848. doi: 10.1038/nrmicro2669 22020261

[B34] Holmes N. A. Walshaw J. Leggett R. M. Thibessard A. Dalton K. A. Gillespie M. D. . (2013). Coiled-coil protein Scy is a key component of a multiprotein assembly controlling polarized growth in *Streptomyces*. Proc. Natl. Acad. Sci. U.S.A. 110, 397–406. doi: 10.1073/pnas.1210657110 23297235 PMC3562780

[B35] Holtmann D. Vernen F. Müller J. M. Kaden D. Risse J. M. Friehs K. . (2017). Effects of particle addition to *Streptomyces* cultivations to optimize the production of actinorhodin and streptavidin. Sustain. Chem. Pharm. 5, 67–71. doi: 10.1016/j.scp.2016.09.001

[B36] Hopwood D. A. (1999). Forty years of genetics with *Streptomyces*: from *in vivo* through *in vitro* to *in silico*. Mikrobiology 145, 2183–2202. doi: 10.1099/00221287-145-9-2183 10517572

[B37] Hsieh Y.-J. Wanner B. L. (2010). Global regulation by the seven-component Pi signaling system. Curr. Opin. Microbiol. 13, 198–203. doi: 10.1016/j.mib.2010.01.014 20171928 PMC2847643

[B38] Jeong Y. Kim J. N. Kim M. W. Bucca G. Cho S. Yoon Y. J. . (2016). The dynamic transcriptional and translational landscape of the model antibiotic producer *Streptomyces coelicolor* A3(2). Nat. Commun. 7, 1–11. doi: 10.1038/ncomms11605 27251447 PMC4895711

[B39] Jiang X. Zghidi-Abouzid O. Oger-Desfeux C. Hommais F. Greliche N. Muskhelishvili G. . (2016). Global transcriptional response of Dickeya dadantii to environmental stimuli relevant to the plant infection. Environ. Microbiol. 18, 3651–3672. doi: 10.1111/1462-2920.13267 26940633

[B40] Kim D. Langmead B. Salzberg S. L. (2015). HISAT: a fast spliced aligner with low memory requirements. Nat. Methods 12, 357–360. doi: 10.1038/nmeth.3317 25751142 PMC4655817

[B41] Kirthika P. Lloren K. K. S. Jawalagatti V. Lee J. H. (2023). Structure, substrate specificity and role of lon protease in bacterial pathogenesis and survival. Internatial J. Mol. Sci. 24:3422. doi: 10.3390/ijms24043422 36834832 PMC9961632

[B42] Kirthika P. Senevirathne A. Jawalagatti V. Park S. W. Lee J. H. (2020). Deletion of the lon gene augments expression of Salmonella Pathogenicity Island (SPI)-1 and metal ion uptake genes leading to the accumulation of bactericidal hydroxyl radicals and host pro-inflammatory cytokine-mediated rapid intracellular clearance. Gut Microbes 11, 1695–1712. doi: 10.1080/19490976.2020.1777923 32567462 PMC7524146

[B43] Kodani S. Hudson M. E. Durrant M. C. Buttner M. J. Nodwell J. R. Willey J. M. (2004). The SapB morphogen is a lantibiotic-like peptide derived from the product of the developmental gene ramS in *Streptomyces coelicolor. Proc. Nat. Acad. Sci*. U.S.A. 101, 11448–11453. doi: 10.1073/pnas.0404220101 PMC509221 15277670

[B44] Koebsch I. Overbeck J. Piepmeyer S. Meschke H. Schrempf H. (2009). A molecular key for building hyphae aggregates: the role of the newly identified *Streptomyces* protein HyaS. Microb. Biotechnol. 2, 343–360. doi: 10.1111/j.1751-7915.2009.00093.x 21261929 PMC3815755

[B45] Kormanec J. Sevcikova B. Novakova R. Homerova D. Rezuchova B. Mingyar E. (2016). “The complex roles and regulation of stress response σ factors in *Streptomyces coelicolor*,” in Stress and Environmental Regulation of Gene Expression and Adaptation in Bacteria, ed. F. J. de Bruijn (Hoboken, NJ: John Wiley & Sons), 328–343. doi: 10.1002/9781119004813.ch29

[B46] Kumar V. Bharti A. Gusain O. Bisht G. S. (2011). Scanning electron microscopy of streptomyces without use of any chemical fixatives. Scanning 33, 446–449. doi: 10.1002/sca.20261 21732388

[B47] Kunst F. Ogasawara N. Moszer I. Albertini A. M. Alloni G. Azevedo V. . (1997). The complete genome sequence of the gram-positive bacterium *Bacillus subtilis*. Nature 390, 249–256. 9384377 10.1038/36786

[B48] Kuroda A. (2006). A polyphosphate-lon protease complex in the adaptation of *Escherichia coli* to amino acid starvation. Biosci. Biotechnol. Biochem. 70, 325–331. doi: 10.1271/bbb.70.325 16495646

[B49] Lajtai-Szabó P. Hülber-Beyer É. Nemestóthy N. Bélafi-Bakó K. (2022). The role of physical support in secondary metabolite production by *Streptomyces* species. Biochem. Eng. J. 185:108495. doi: 10.1016/j.bej.2022.108495

[B50] Langmead B. Salzberg S. L. (2012). Fast gapped-read alignment with Bowtie 2. Nat. Methods 9, 357–359. doi: 10.1038/nmeth.1923 22388286 PMC3322381

[B51] Lee E. Karoonuthaisiri N. Kim H. Park J. Cha C. Kao C. M. . (2005). A master regulator σB governs osmotic and oxidative response as well as differentiation via a network of sigma factors in *Streptomyces coelicolor*. Mol. Microbiol. 57, 1252–1264. doi: 10.1111/j.1365-2958.2005.04761.x 16101999

[B52] Lee I. Suzuki C. K. (2008). Functional mechanics of the ATP-dependent Lon protease- lessons from endogenous protein and synthetic peptide substrates. Biochim. Biophys. Acta Proteins Proteomics 1784, 727–735. doi: 10.1016/j.bbapap.2008.02.010 18359303 PMC2443057

[B53] Lewis R. A. Laing E. Allenby N. Bucca G. Brenner V. Harrison M. . (2010). Metabolic and evolutionary insights into the closely-related species *Streptomyces coelicolor* and *Streptomyces lividans* deduced from high-resolution comparative genomic hybridization. BMC Genom. 11, 1–16. doi: 10.1186/1471-2164-11-682 21122120 PMC3017869

[B54] Li B. Dewey C. N. (2011). RSEM: accurate transcript quantification from RNA-Seq data with or without a reference genome. BMC Bioinformatics 12:323. doi: 10.1186/1471-2105-12-323 21816040 PMC3163565

[B55] Li H. Durbin R. (2009). Fast and accurate short read alignment with Burrows–Wheeler transform. Bioinformatics 25, 1754–1760. doi: 10.1093/bioinformatics/btp324 19451168 PMC2705234

[B56] Li H. Handsaker B. Wysoker A. Fennell T. Ruan J. Homer N. . (2009). The sequence alignment/map format and SAMtools. Bioinformatics 25, 2078–2079. doi: 10.1093/bioinformatics/btp352 19505943 PMC2723002

[B57] Liu G. Chater K. F. Chandra G. Niu G. Tan H. (2013). Molecular regulation of antibiotic biosynthesis in streptomyces. Microbiol. Mol. Biol. Rev. 77, 112–143. doi: 10.1128/MMBR.00054-12 23471619 PMC3591988

[B58] Liu J. Cosby W. M. Zuber P. (1999). Role of Lon and ClpX in the post-translational regulation of a sigma subunit of RNA polymerase required for cellular differentiation in *Bacillus subtilis*. Mol. Microbiol. 33, 415–428. doi: 10.1046/j.1365-2958.1999.01489.x 10411757

[B59] Livak K. J. Schmittgen T. D. (2001). Analysis of relative gene expression data using real-time quantitative PCR and the 2^−ΔΔ*CT*^ method. Methods 25, 402–408. doi: 10.1006/meth.2001.1262 11846609

[B60] Loehlin D. W. Carroll S. B. (2016). Expression of tandem gene duplicates is often greater than twofold. Proc. Natl. Acad. Sci. U.S.A. 113, 5988–5992. doi: 10.1073/pnas.1605886113 27162370 PMC4889415

[B61] López-García M. T. Rioseras B. Yagüe P. Álvarez J. R. Manteca Á. (2014). Cell immobilization of *Streptomyces coelicolor*: effect on differentiation and actinorhodin production. Int. Microbiol. 17, 75–80. doi: 10.2436/20.1501.01.209 26418851 PMC4597334

[B62] Love M. I. Huber W. Anders S. (2014). Moderated estimation of fold change and dispersion for RNA-seq data with DESeq2. Genome Biol. 15:550. doi: 10.1186/s13059-014-0550-8 25516281 PMC4302049

[B63] Lu Y. Wang W. Shu D. Zhang W. Chen L. Qin Z. . (2007). Characterization of a novel two-component regulatory system involved in the regulation of both actinorhodin and a type I polyketide in *Streptomyces coelicolor*. Appl. Microbiol. Biotechnol. 77, 625–635. doi: 10.1007/s00253-007-1184-5 17899070

[B64] Manteca A. Alvarez R. Salazar N. Yagüe P. Sanchez J. (2008). Mycelium differentiation and antibiotic production in submerged cultures of *Streptomyces coelicolor*. Appl. Environ. Microbiol. 74, 3877–3886. doi: 10.1128/AEM.02715-07 18441105 PMC2446541

[B65] Manteca A. Jung H. R. Schwammle V. Jensen O. N. Sanchez J. (2010a). Quantitative proteome analysis of *Streptomyces coelicolor* nonsporulating liquid cultures demonstrates a complex differentiation process comparable to that occurring in sporulating solid cultures. J. Proteome Res. 9, 4801–4811. doi: 10.1021/pr100513p 20681593

[B66] Manteca A. Sanchez J. Jung H. R. Schwämmle V. Jensen O. N. (2010b). Quantitative proteomics analysis of *Streptomyces coelicolor* development demonstrates that onset of secondary metabolism coincides with hypha differentiation. Mol. Cell. Proteomics 9, 1423–1436. doi: 10.1074/mcp.M900449-MCP200 20224110 PMC2938082

[B67] Manteca Á. Yagüe P. (2018). *Streptomyces* differentiation in liquid cultures as a trigger of secondary metabolism. Antibiotics 7:41. doi: 10.3390/antibiotics7020041 29757948 PMC6022995

[B68] Marr A. K. Overhage J. Bains M. Hancock R. E. W. (2007). The Lon protease of Pseudomonas aeruginosa is induced by aminoglycosides and is involved in biofilm formation and motility. Microbiol. 153, 474–482. doi: 10.1099/mic.0.2006/002519-0 17259618

[B69] Martín J. F. (2004). Phosphate control of the biosynthesis of antibiotics and other secondary metabolites is mediated by the PhoR-PhoP system: an unfinished story. J. Bacteriol. 186, 5197–5201. doi: 10.1128/JB.186.16.5197-5201.2004 15292120 PMC490900

[B70] Martín J. F. Liras P. (2021). Molecular mechanisms of phosphate sensing, transport and signalling in streptomyces and related actinobacteria. Int. J. Mol. Sci. 22, 1–20. doi: 10.3390/ijms22031129 33498785 PMC7866108

[B71] Martín J. F. Santos-Beneit F. Rodríguez-García A. Sola-Landa A. Smith M. C. M. Ellingsen T. E. . (2012). Transcriptomic studies of phosphate control of primary and secondary metabolism in *Streptomyces coelicolor*. Appl. Microbiol. Biotechnol. 95, 61–75. doi: 10.1007/s00253-012-4129-6 22622839

[B72] McKenna A. Hanna M. Banks E. Sivachenko A. Cibulskis K. Kernytsky A. . (2010). The Genome Analysis Toolkit: a MapReduce framework for analyzing next-generation DNA sequencing data. Genome Res. 20, 1297–1303. doi: 10.1101/gr.107524.110 20644199 PMC2928508

[B73] McLaren W. Gil L. Hunt S. E. Riat H. S. Ritchie G. R. S. Thormann A. . (2016). The ensembl variant effect predictor. Genome Biol. 17:122. doi: 10.1186/s13059-016-0974-4 27268795 PMC4893825

[B74] Merrick M. J. (1976). A morphological and genetic mapping study of bald colony mutants of *Streptomyces coelicolor*. Microbiology 96, 299–315. doi: 10.1099/00221287-96-2-299 186556

[B75] Mizusawa S. Gottesman S. (1983). Protein degradation in *Escherichia coli*: the lon gene controls the stability of sulA protein. Proc. Nat. Acad. Sci. U.S.A. 80, 358–362. doi: 10.1073/pnas.80.2.358 6300834 PMC393376

[B76] Nodwell J. R. McGovern K. Losick R. (1996). An oligopeptide permease responsible for the import of an extracellular signal governing aerial mycelium formation in *Streptomyces coelicolor*. Mol. Microbiol. 22, 881–893. doi: 10.1046/j.1365-2958.1996.01540.x 8971710

[B77] O'Connor T. J. Nodwell J. R. (2005). Pivotal roles for the receiver domain in the mechanism of action of the response regulator RamR of *Streptomyces coelicolor*. J. Mol. Biol. 351, 1030–1047. doi: 10.1016/j.jmb.2005.06.053 16051268

[B78] Omnus D. J. Fink M. J. Szwedo K. Jonas K. (2021). The Lon protease temporally restricts polar cell differentiation events during the *Caulobacter* cell cycle. Elife. 10,1–28. doi: 10.7554/eLife.73875 34693909 PMC8545394

[B79] Ou X. Zhang B. Zhang L. Dong K. Liu C. Zhao G. . (2008). SarA influences the sporulation and secondary metabolism in *Streptomyces coelicolor* M145. Acta Biochim. Biophys. Sin. 40, 877–882. doi: 10.1111/j.1745-7270.2008.00466.x 18850053

[B80] Potúčková L. Kelemen G. H. Findlay K. C. Lonetto M. A. Buttner M. J. Kormanec J. (1995). A new RNA polymerase sigma factor, σF is required for the late stages of morphological differentiation in *Streptomyces* spp. Mol. Microbiol. 17, 37–48. doi: 10.1111/j.1365-2958.1995.mmi_17010037.x 7476207

[B81] Rigali S. Titgemeyer F. Barends S. Mulder S. Thomae A. W. Hopwood D. A. . (2008). Feast or famine: the global regulator DasR links nutrient stress to antibiotic production by *Streptomyces*. Sci. Rep. 9, 670–675. doi: 10.1038/embor.2008.83 18511939 PMC2475330

[B82] Saito A. Shinya T. Miyamoto K. Yokoyama T. Kaku H. Minami E. . (2007). The dasABC gene cluster, adjacent to dasR, encodes a novel ABC transporter for the uptake of N,N′-diacetylchitobiose in *Streptomyces coelicolor* A3(2). *Appl. Environ. Microbiol*. 73, 3000–3008. doi: 10.1128/AEM.02612-06 PMC1892892 17351098

[B83] Sánchez de la Nieta R. Antoraz S. Alzate J. F. Santamaría R. I. Díaz M. (2020). Antibiotic production and antibiotic resistance: the two sides of AbrB1/B2, a two-component system of *Streptomyces coelicolor*. Front. Microbiol. 11:587750. doi: 10.3389/fmicb.2020.587750 33162964 PMC7581861

[B84] Santos-Beneit F. Rodríguez-García A. Franco-Domínguez E. Martín J. F. (2008). Phosphate-dependent regulation of the low- and high-affinity transport systems in the model actinomycete *Streptomyces coelicolor*. Microbiology 1, 2356–2370. doi: 10.1099/mic.0.2008/019539-0 18667568

[B85] Schmid M. B. Roth J. R. (1987). Gene location affects expression level in *Salmonella typhimurium*. J. Bacteriol. 169, 2872–2875. doi: 10.1128/jb.169.6.2872-2875.1987 3294809 PMC212203

[B86] Seghezzi N. Darbon E. Martel C. David M. Lejeune C. Esnault C. . (2022). The generation of an artificial atp deficit triggers antibiotic production in *Streptomyces lividans*. Antibiotics 11:1157. doi: 10.3390/antibiotics11091157 36139937 PMC9495134

[B87] Silva F. Queiroz J. A. Domingues F. C. (2012). Evaluating metabolic stress and plasmid stability in plasmid DNA production by *Escherichia coli*. Biotechnol. Adv. 30, 691–708. doi: 10.1016/j.biotechadv.2011.12.005 22244816

[B88] Sobczyk A. Bellier A. Viala J. Mazodier P. (2002). The *lon* gene, encoding an ATP-dependent protease, is a novel member of the HAIR/HspR stress-response regulon in actinomycetes. Microbiol. Soc. 148, 1931–1933. doi: 10.1099/00221287-148-6-1931 12055312

[B89] Sohoni S. V. Bapat P. M. Lantz A. E. (2012). Robust, small-scale cultivation platform for *Streptomyces coelicolor*. Microb. Cell Fact. 11:9. doi: 10.1186/1475-2859-11-9 22252012 PMC3292921

[B90] Sola-Landa A. Moura R. S. Martín J. F. (2003). The two-component PhoR-PhoP system controls both primary metabolism and secondary metabolite biosynthesis in *Streptomyces lividans*. Proc. Nat. Acad. Sci. U.S.A. 100, 6133–6138. doi: 10.1073/pnas.0931429100 12730372 PMC156338

[B91] Sprusansky O. Stirrett K. Skinner D. Denoya C. Westpheling J. (2005). The bkdR gene of *Streptomyces coelicolor* is required for morphogenesis and antibiotic production and encodes a transcriptional regulator of a branched-chain amino acid dehydrogenase complex. J. Bacteriol. 187, 664–671. doi: 10.1128/JB.187.2.664-671.2005 15629937 PMC543559

[B92] Stirrett K. Denoya C. Westpheling J. (2009). Branched-chain amino acid catabolism provides precursors for the Type II polyketide antibiotic, actinorhodin, via pathways that are nutrient dependent. J. Ind. Microbiol. Biotechnol. 36, 129–137. doi: 10.1007/s10295-008-0480-0 18841403

[B93] StrepDB (2025). SCO3793 Genomic Region Viewer. Available online at: https://strepdb.streptomyces.org.uk/cgi-bin/dc3.pl?accession=AL645882andserial=3778andwidth=1200andstart=4166510andend=4176510andiorm=map (Accessed November 30, 2025).

[B94] Świa̧tek-Połatyńska M. A. Bucca G. Laing E. Gubbens J. Titgemeyer F. Smith C. P. . (2015). Genome-wide analysis of in vivo binding of the master regulator DasR in *Streptomyces coelicolor* identifies novel non-canonical targets. PLoS ONE 10:e0122479. doi: 10.1371/journal.pone.0122479 25875084 PMC4398421

[B95] Takaya A. Tabuchi F. Tsuchiya H. Isogai E. Yamamoto T. (2008). Negative regulation of quorum-sensing systems in *Pseudomonas aeruginosa* by ATP-dependent lon protease. J. Bacteriol. 190, 4181–4188. doi: 10.1128/JB.01873-07 18408026 PMC2446771

[B96] Team R. S. (2021). RStudio: Integrated Development Environment for R. Boston, MA: RStudio.

[B97] Thompson A. Gasson M. J. (2001). Location effects of a reporter gene on expression levels and on native protein synthesis in *Lactococcus lactis* and *Saccharomyces cerevisiae*. Appl. Environ. Microbiol. 67, 3434–3439. doi: 10.1128/AEM.67.8.3434-3439.2001 11472915 PMC93039

[B98] Tojo N. Inouye S. Komano T. (1993). The *lon*D gene is homologous to the lon gene encoding an ATP-dependent protease and is essential for the development of *Myxococcus xanthus*. J. Bacteriol. 175, 4545–4549. doi: 10.1128/jb.175.14.4545-4549.1993 8331083 PMC204897

[B99] Torres-Cabassa A. S. Gottesman S. (1987). Capsule synthesis in *Escherichia coli* K-12 is regulated by proteolysis. J. Bacteriol. 169, 981–989. doi: 10.1128/jb.169.3.981-989.1987 3029041 PMC211890

[B100] Traag B. A. van Wezel G. P. (2008). The SsgA-like proteins in actinomycetes: small proteins up to a big task. Antonie Van Leeuwenhoek 94, 85–97. doi: 10.1007/s10482-008-9225-3 18273689 PMC2440963

[B101] Tsilibaris V. Maenhaut-Michel G. Van Melderen L. (2006). Biological roles of the Lon ATP-dependent protease. Res. Microbiol. 157, 701–713. doi: 10.1016/j.resmic.2006.05.004 16854568

[B102] Van Dissel D. Claessen D. van Wezel G. P. (2014). Morphogenesis of streptomyces in submerged cultures. Adv. Appl. Microbiol. 89, 1–45. doi: 10.1016/B978-0-12-800259-9.00001-9 25131399

[B103] Van Veluw G. J. Petrus M. L. C. Gubbens J. De Graaf R. De Jong I. P. Van Wezel G. P. . (2012). Analysis of two distinct mycelial populations in liquid-grown *Streptomyces* cultures using a flow cytometry-based proteomics approach. *Appl. Microbiol*. Biotechnol. 96, 1301–1312. doi: 10.1007/s00253-012-4490-5 23070651

[B104] Van Wezel G. P. Krabben P. Traag B. A. Keijser B. J. F. Kerste R. Vijgenboom E. . (2006). Unlocking *Streptomyces* spp. for use as sustainable industrial production platforms by morphological engineering. Appl. Environ. Microbiol. 72, 5283–5288. doi: 10.1128/AEM.00808-06 16885277 PMC1538695

[B105] Van Wezel G. P. McDowall K. J. (2011). The regulation of the secondary metabolism of streptomyces: new links and experimental advances. Nat. Prod. Rep. 28, 1311–1333. doi: 10.1039/c1np00003a 21611665

[B106] Virolle M.-J. (2020). A challenging view: antibiotics play a role in the regulation of the energetic metabolism of the producing bacteria. Antibiotics 9:83. doi: 10.3390/antibiotics9020083 32069930 PMC7168255

[B107] Vollmer B. Steblau N. Ladwig N. Mayer C. Macek B. Mitousis L. . (2019). Role of the *Streptomyces* spore wall synthesizing complex SSSC in differentiation of *Streptomyces coelicolor* A3(2). *Int. J. Med. Microbiol*. 309:151327. doi: 10.1016/j.ijmm.2019.07.001 31324525

[B108] Wang Z. Xiang L. Shao J. Wegrzyn A. Wegrzyn G. (2006). Effects of the presence of ColE1 plasmid DNA in *Escherichia coli* on the host cell metabolism. Microbial Cell Factory 5:34. doi: 10.1186/1475-2859-5-34 17112383 PMC1664580

[B109] Whistler C. A. Stockwell V. O. Loper J. E. (2000). Lon protease influences antibiotic production and UV tolerance of *Pseudomonas fluorescens* Pf-5. Appl. Environ. Microbiol. 66, 2718–2725. doi: 10.1128/AEM.66.7.2718-2725.2000 10877760 PMC92065

[B110] Wright R. Stephens C. Zweiger G. Shapiro L. Alley M. R. (1996). Caulobacter Lon protease has a critical role in cell-cycle control of DNA methylation. Genes Dev. 10, 1532–1542. doi: 10.1101/gad.10.12.1532 8666236

[B111] Yagüe P. Manteca A. Simon A. Diaz-Garcia M. E. Sanchez J. (2010). New method for monitoring programmed cell death and differentiation in submerged *Streptomyces* cultures. Appl. Environ. Microbiol. 76, 3401–3404. doi: 10.1128/AEM.00120-10 20348294 PMC2869136

[B112] Yagüe P. Rodríguez-García A. López-García M. T. Martín J. F. Rioseras B. Sánchez J. . (2013). Transcriptomic analysis of *Streptomyces coelicolor* differentiation in solid sporulating cultures: first compartmentalized and second multinucleated mycelia have different and distinctive transcriptomes. PLoS ONE 8:e60665. doi: 10.1371/journal.pone.0060665 23555999 PMC3610822

[B113] Yagüe P. Rodríguez-García A. López-García M. T. Rioseras B. Martín J. F. Sánchez J. . (2014). Transcriptomic analysis of liquid non-sporulating *Streptomyces coelicolor* cultures demonstrates the existence of a complex differentiation comparable to that occurring in solid sporulating cultures. PLoS ONE 9:e86296. doi: 10.1371/journal.pone.0086296 24466012 PMC3897704

[B114] Yilmaz H. Yaradir E. Tunca S. (2025). Expression of multiple copies of the lon protease gene resulted in increased antibiotic production, osmotic and UV stress resistance in *Streptomyces coelicolor* A3(2). *Curr. Microbiol*. 82:43. doi: 10.1007/s00284-024-04021-z 39690306

[B115] Yong-quan L. Pei-lin C. Shi-fei C. Dan W. Jing Z. (2004). A pair of two-component regulatory genes ecr A1/A2 in *S. coelicolor*. J. Zhejiang Univ. Sci. 5, 173–179. doi: 10.1631/BF02840919 14674028

[B116] Zhang J. Liang Q. Xu Z. Cui M. Zhang Q. Abreu S. . (2020). The inhibition of antibiotic production in *Streptomyces coelicolor* over-expressing the TetR regulator SCO3201 is correlated with changes in the lipidome of the strain. Front. Microbiol. 11:1399. doi: 10.3389/fmicb.2020.01399 32655536 PMC7324645

