# Supplementary material for: Lon protease reprograms cellular physiology of Streptomyces coelicolor resulting in enhance antibiotic production
Source: Front Microbiol. 2026 Mar 16;17:1789434. doi: 10.3389/fmicb.2026.1789434 (PMC13033682; doi:10.3389/fmicb.2026.1789434)
Supplement: Supplementary file 1 [file Data_Sheet_1.docx]

Supplementary Material


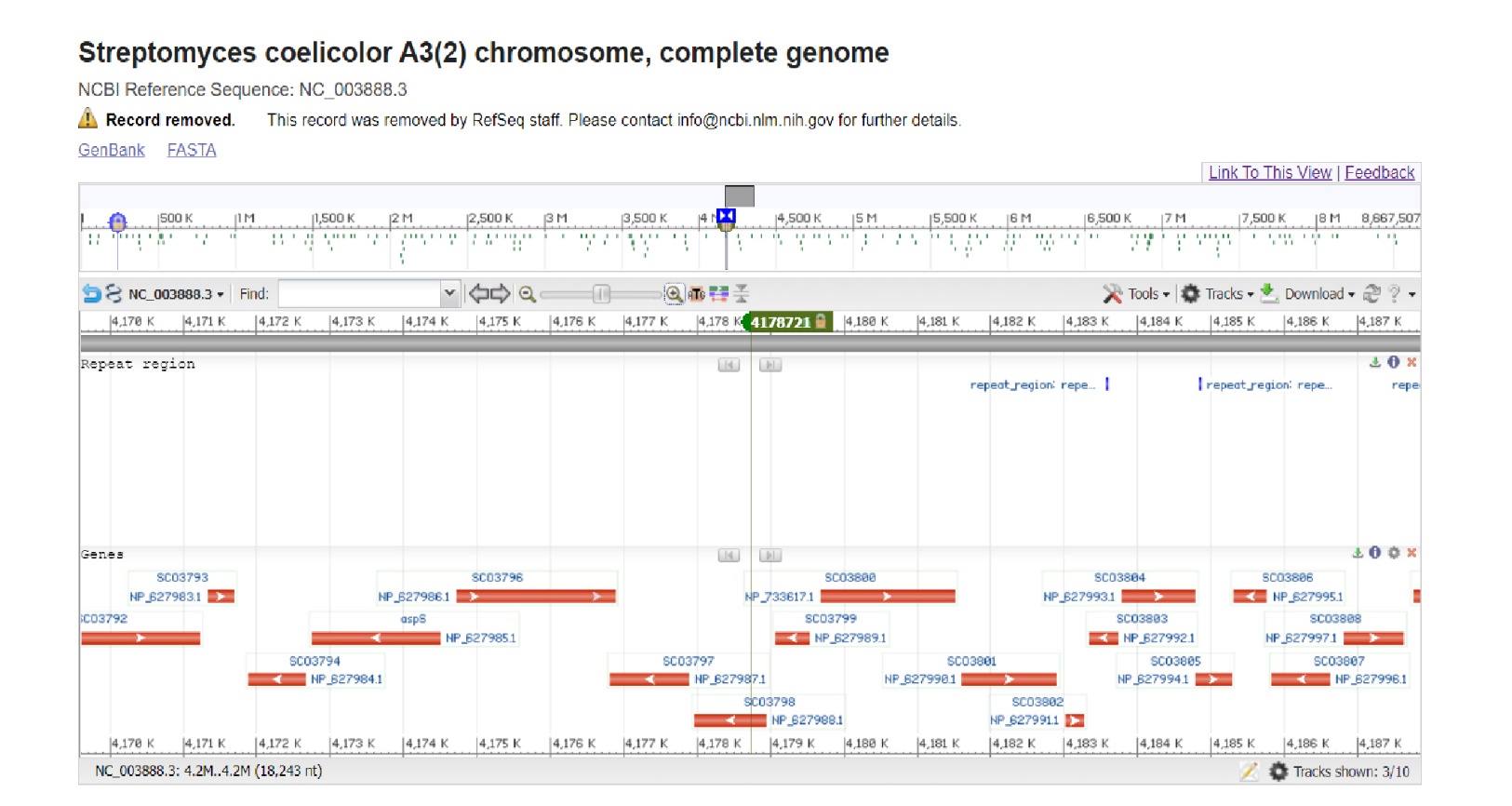
Supplementary Figures

**Supplementary Fig 1.** Schematic representation of the genomic region where the pRA vector is integrated into the *Streptomyces coelicolor* A3(2) genome. The integration site within the SCO3798 gene is indicated by a green line (<https://go.usa.gov/xeE5V> )


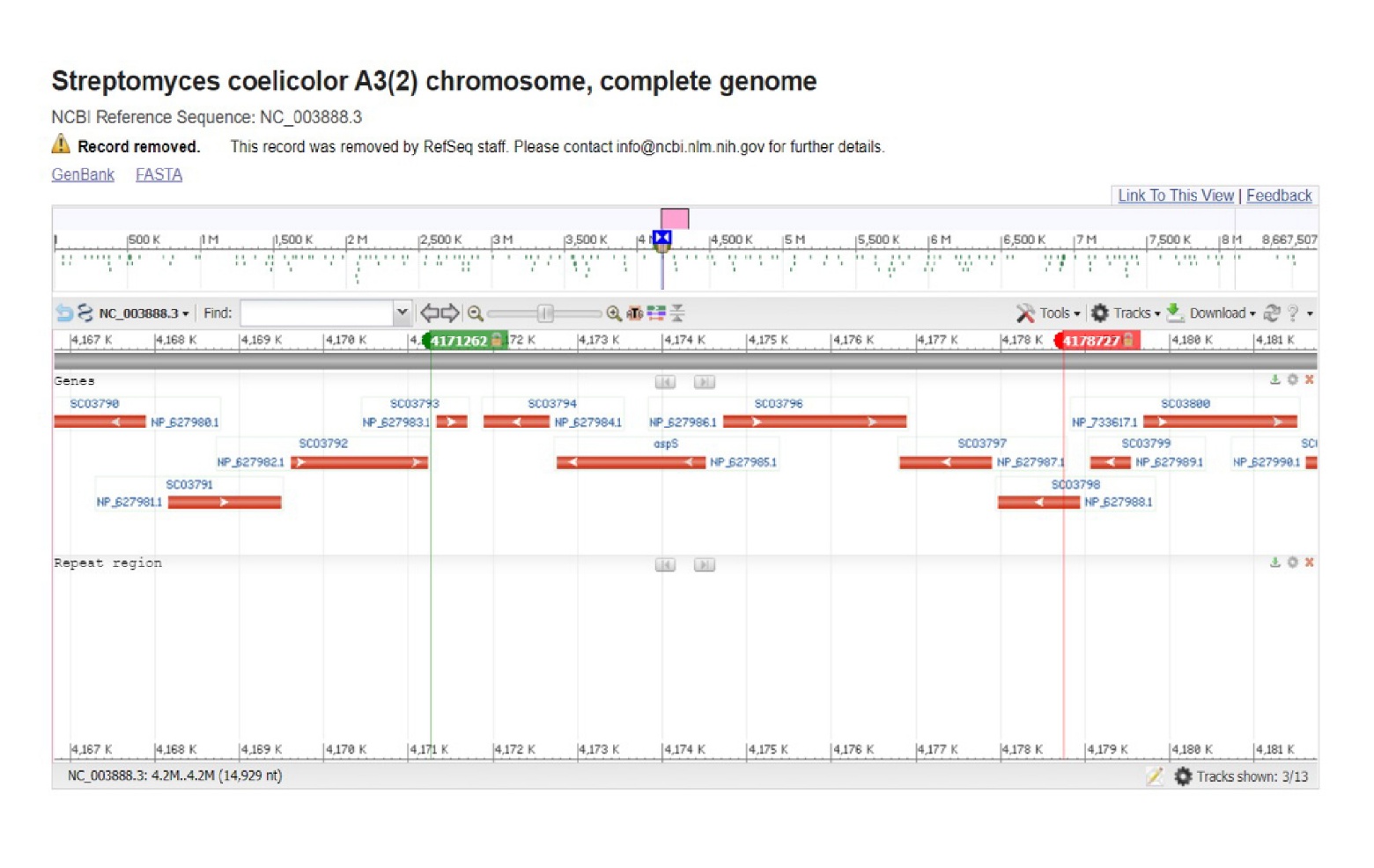
**Supplementary Fig 2.** Schematic representation of the genomic regions where the pRA-*lon* vector is integrated into the *Streptomyces coelicolor* A3(2) genome. Two integration sites within the SCO3793 and SCO3798 genes are indicated by green and red lines, respectively (<https://go.usa.gov/xfKaG>).

**
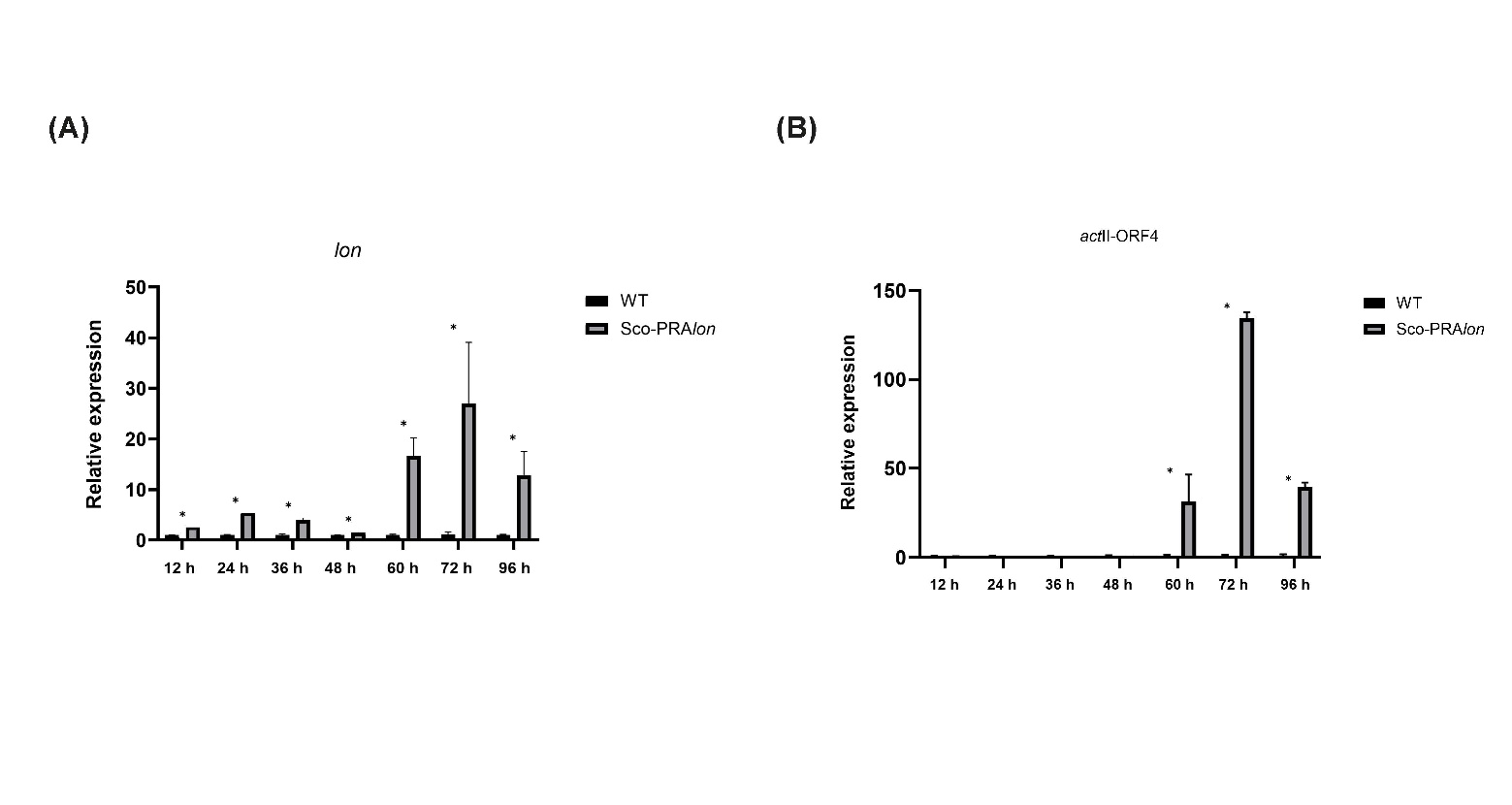
****Supplementary Fig 3.** Relative gene expression of *lon* (A) and *act*II-ORF4 (B) genes in the Sco-pRA*lon* strain compared to the wild-type (WT) at different timepoints. The statistical significance analysis is performed using Prism 8.0.2 software (GraphPad). *P-*values were calculated using the Mann–Whitney test. Asterisks (*) represent a *p-value* of <0.05. Vertical bars indicate the standard deviation from the mean value of two biological replicates.


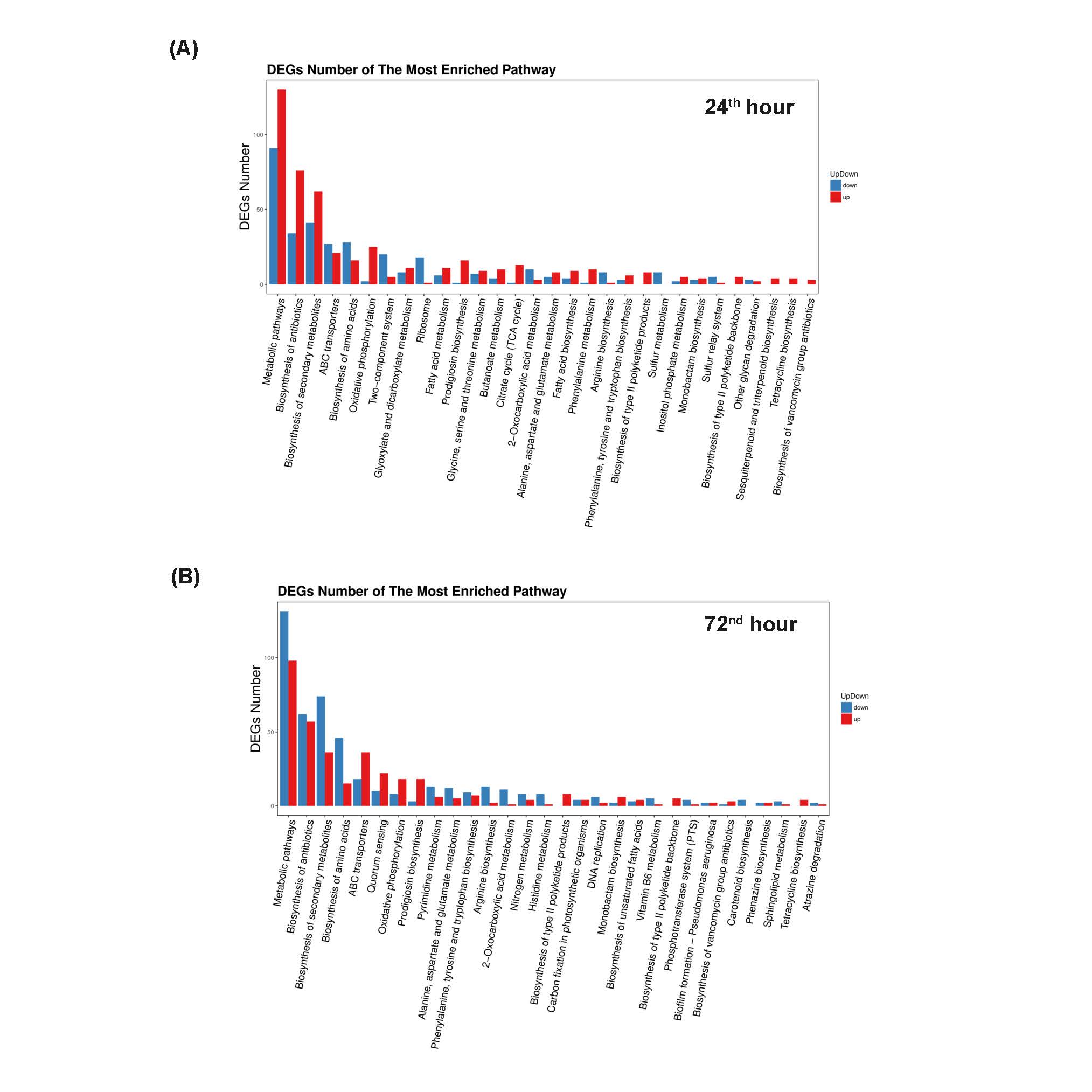
**Supplemantary Fig 4.**Significantly up or downregulated DEGs of the enriched KEGG pathways at 24^th^ (A) and 72^nd^ (B) hours. The X-axis represents pathway name and the Y -axis represents the number of DEGs.
