# Supplementary material for: Lon protease reprograms cellular physiology of Streptomyces coelicolor resulting in enhance antibiotic production
Source: Front Microbiol. 2026 Mar 16;17:1789434. doi: 10.3389/fmicb.2026.1789434 (PMC13033682; doi:10.3389/fmicb.2026.1789434)
Supplement: Supplementary file 3 [file Table_2.docx]

**Supplementary Table S2** Primers used in qRT-PCR.

| Primer | Sequence (5’-3’) | Amplicon size (bp) | Target gene |
| --- | --- | --- | --- |
| Determination of samples for RNA-seq | | | |
| SCO5285-F | AGCTGGTGAAGGAGTACA TCAGGAACGGCGAGTAG | 135 | *lon* |
| SCO5285-R |  |  |  |
| SC05085-F | GAGCTGCGGCTTTTTGGAAT TGCGAAATTACCAGGGACCG | 101 | *actII*-ORF4 |
| SCO5085-R |  |  |  |
| RT-qPCR validation of RNA-Seq | | | |
| SCO0379-F | AGCAGGGCATCAAGAACCTC  GGTGGAAGCGGTAGTTCTCC | 171 | *cat*A |
| SCO0379-R |  |  |  |
| SCO4659-F | TCGAGAAGAACAAGACGCCC  CGGAATGTAAGCGGTGACCT | 158 | *rps*L |
| SCO4659-R |  |  |  |
| SCO2518-F | TCATCGCCATCCTCTTCTGC  GATGAGGGTGATGCTGTGCG | 185 | *ecra*2 |
| SCO2518-R |  |  |  |
| SCO5074-F | CGGGCAAGAAGTTCAAGG  GTCGTTGACGATCTGGTTG | 77 | *act*VI-3 |
| SCO5074-R |  |  |  |
| SCO6214-F | TG CATGAAGCCGTTGAAGACCG  GTGATGGTGGTCTCGATG | 152 | putative permease |
| SCO6214-R |  |  |  |
| SCO0409-F | CGGCTACAAGGTGGTGAACT  ATGGTCACGGTGCAGTTCTT | 96 | *sap*A |
| SCO0409-R |  |  |  |
| SCO4139-F | GACGACGAGAACCTCTACGG  TTGTCGTACACCGACATCGT | 110 | *pts*B |
| SCO4139-R |  |  |  |
| SCO0600-F | GGAGTTGTCGAAGCTGTT CTT  GTTCATCTCGATCAGCGTGTT | 110 | *sig*B |
| SCO0600-R |  |  |  |
| SCO4878-F | CGATCATCTCCCACCATCTC  CGCTCATCGCCTTCAGTT | 75 | putative glycosyltransferase |
| SCO4878-R |  |  |  |
| SCO5893-F | GCAAGGACTTCGTGATCGAG  GCGTTGGAGACGTAGAGCAG | 103 | *red*K |
| SCO5893-R |  |  |  |
| SCO6577-F | GCTCGGCTTCTCGGTCTATC  GAGTTCACCAGGACCTGCTC | 96 | conserved hypothetical protein SC3F9.12 |
| SCO6577-R |  |  |  |
| SCO1675-F  SCO1675-R | AACGTCGTTCAGGTTCCCG  GGGTTCAGCAGACCGATCAC | 77 | *chp*H |
| SCO1675-R  SCO1675-R |  |  |  |
| Reference Genes | | |  |
| SCO3873-F | GGTACACCGAGTGCAAGAT  GTCGT GTTGTCCGTGAAGT | 89 | *gyr*A |
| SCO3873-R |  |  |  |
| SCO5820-F | TGCTCTTCCTGGACCTCAT  GAACTTGTAGCCCTTGGTGTAG | 86 | *hrd*B |
| SCO5820-R |  |  |  |

F* forward; R* reverse
